# Copper-Iron Cell Death Axis: Mechanistic Crosstalk, Disease Implications and an Integrated Metallo-Redox-Metabolic Framework

**DOI:** 10.7150/ijbs.132221

**Published:** 2026-05-22

**Authors:** HaoQi Huang, YuLu Chen, Yi Lu, ZiLong Yuan, Ahmed Zahoor, LiPing Ren, YuanYuan Luo, BaoCai Zhong, Jian Huang, Hui Chen, Lin Ning

**Affiliations:** 1 School of Life Science and Technology, University of Electronic Science and Technology of China, Chengdu, Sichuan, China.; 2 School of Medical Technology and Information Engineering, Zhejiang Chinese Medical University, Hangzhou, Zhejiang, China.; 3 School of Healthcare and Technology, Chengdu Neusoft University, Chengdu, Sichuan, China.; 4 Yangtze Delta Region Institute (Quzhou), University of Electronic Science and Technology of China, Quzhou, Zhejiang, China.

**Keywords:** metal-dependent programmed cell death, metallo-redox-metabolic framework, cuproptosis, ferroptosis, bioinformatics

## Abstract

Cuproptosis and ferroptosis are two major forms of metal-dependent cell death, characterized by mitochondrial proteotoxicity and lipid peroxidation, respectively, and are broadly implicated in diverse disease contexts. Here, by integrating mechanistic, biological, and disease-associated evidence, we propose the metal-metabolism-redox vulnerability axis, which describes cellular states under metal stress as a continuous space defined by metal homeostasis, mitochondrial metabolism, and redox balance. Within this space, cuproptosis and ferroptosis correspond to distinct execution regions rather than independent processes. Building on this concept, we further establish a metallo-redox-metabolic framework to explain how key state variables and their coupling relationships determine execution bias and drive dynamic transitions between death modalities. This framework reframes metal-dependent cell death as a state-driven system rather than a collection of discrete pathways and provides a unified perspective for understanding its roles in complex diseases. In addition, we outline predictive and testable hypotheses and highlight the importance of multi-omics integration and artificial intelligence based modeling in capturing cellular state and enabling dynamic prediction. Collectively, this work provides a conceptual foundation for understanding metal-driven cell fate decisions and for developing state-oriented therapeutic strategies.

## Introduction

Programmed cell death (PCD) is fundamental to the maintenance of tissue homeostasis, and its dysregulation is implicated in a broad spectrum of pathological conditions, including cancer, neurodegenerative disorders, as well as chronic inflammatory and metabolic diseases 1-3. In recent years, emerging forms of metal-dependent cell death have substantially expanded our understanding of how essential trace metals regulate mitochondrial metabolism, redox balance, and cellular vulnerability 4, 5. Among these, ferroptosis is characterized by iron-dependent lipid peroxidation 6, whereas cuproptosis is driven by copper-induced aggregation of lipoylated mitochondrial enzymes 7. These two forms of cell death were initially considered mechanistically distinct. However, accumulating evidence suggests that they share common upstream determinants, including disruption of metal homeostasis 8, increased mitochondrial metabolic burden, and instability of iron-sulfur (Fe-S) cluster systems. These findings indicate that copper- and iron-dependent cell death may not represent fully independent processes, but instead arise from a more integrated biological stress system, giving rise to distinct death outcomes under different cellular contexts 9, 10. Notably, in complex diseases such as cancer and neurodegeneration, metal imbalance, metabolic stress, and redox dysregulation frequently coexist and interact 7, 11, further supporting the presence of a deeper mechanistic connection between ferroptosis and cuproptosis. Collectively, these observations highlight the need to re-examine metal-dependent cell death from a systems-level perspective.

In light of these considerations, we propose that ferroptosis and cuproptosis can be more appropriately understood as distinct manifestations within a shared biological continuum, which we term the metal-metabolism-redox vulnerability axis. This axis represents a cellular state space defined by the interplay of metal flux, mitochondrial metabolic load, and redox capacity, which together determine the overall susceptibility of cells to metal-induced stress. Within this conceptualization, ferroptosis and cuproptosis are no longer viewed simply as parallel and independent pathways, but rather as distinct execution outcomes driven by different state configurations along this common vulnerability axis.

Building upon this biologically grounded perspective, we further propose a metallo-redox-metabolic framework as a conceptual model to systematically organize, interpret, and predict how dynamic perturbations within the vulnerability axis lead to specific cell death outcomes. Within this framework, key factors—including metal homeostasis, metabolic activity, and redox balance—are integrated to map distinct cellular states to corresponding execution biases in cell death modalities. From this perspective, metal-dependent cell death can be understood as a dynamic, systems-level process, in which ferroptosis and cuproptosis emerge from the coordinated integration of upstream stress signals across multiple regulatory layers, rather than being dictated by isolated molecular pathways.

Importantly, this framework enables the formulation of predictive and testable hypotheses. For instance, it provides a basis for investigating how different positions along the vulnerability axis bias cells toward specific death modalities, how transitions between these states may occur, and how such dynamics vary across biological contexts. Moreover, the proposed metallo-redox-metabolic framework offers a structured and testable foundation for understanding metal-driven cell fate decisions, while also providing a clear rationale for subsequent computational modeling and experimental validation. By shifting from static descriptions toward dynamic and integrative interpretation, this framework opens new avenues for precision intervention strategies targeting upstream vulnerability states in complex diseases 12.

## Copper- and Iron-Dependent Cell Death: Core Biological Features

Copper- and iron-dependent forms of regulated cell death have markedly advanced our understanding of how essential metal ions shape cellular vulnerability 13, 14. Ferroptosis is an iron-dependent oxidative death modality driven by phospholipid peroxidation, whereas cuproptosis is initiated by copper-induced aggregation of lipoylated mitochondrial enzymes and collapse of mitochondrial proteostasis 15-17. Despite their distinct biochemical triggers, both modalities belong to a broader class of non-apoptotic, metabolism-centered cell death programs governed by metal-dependent stress rather than caspase-mediated execution 18. Their initiation and progression are dictated by the interplay between metal homeostasis, mitochondrial metabolic load and redox architecture 19, creating a biochemical landscape in which subtle shifts in metabolic configuration or metal availability can redirect cellular fate. These considerations motivate a detailed examination of the molecular mechanisms underlying cuproptosis and ferroptosis.

### Mechanism of Cuproptosis

Cuproptosis is a mitochondria-centered form of regulated cell death, initiated by aberrant interactions between reduced copper and a specific subset of lipoylated mitochondrial enzyme complexes that serve as structural and functional anchors of the tricarboxylic acid (TCA) cycle 7. In cellular states characterized by high TCA flux and active oxidative phosphorylation, these lipoylated dehydrogenase complexes provide a preferential biochemical interface for copper coordination, thereby conferring a metabolism-dependent susceptibility to copper-induced stress 20, 21.

At the molecular level, the initiation of cuproptosis involves the reduction of Cu²⁺ to Cu⁺ mediated by FDX1 and related redox-active factors, followed by high-affinity binding of Cu⁺ to lipoylated proteins such as DLAT 22. This interaction promotes aberrant protein crosslinking and oligomerization, leading to the progressive accumulation of insoluble aggregates within the mitochondrial matrix 23. As proteotoxic stress intensifies, mitochondrial chaperone systems and proteostasis networks become overwhelmed, ultimately resulting in collapse of matrix protein homeostasis. This primary proteotoxic insult directly disrupts the function of TCA-associated enzymes and interferes with coordinated metabolic regulation, thereby generating a progressively amplifying mitochondrial burden 24, 25.

Building upon this primary damage, mitochondrial dysfunction further propagates into a series of secondary, system-level failures, among which impairment of iron-sulfur (Fe-S) cluster-containing proteins represents a critical amplifying process. Importantly, Fe-S proteins should not be viewed as initial targets of copper toxicity; rather, their dysfunction emerges progressively in the context of sustained proteotoxic stress and mitochondrial destabilization. The Fe-S proteome is functionally heterogeneous, encompassing (i) metabolic enzymes involved in TCA-cycle flux (e.g., aconitase), (ii) respiratory chain components that support electron transport, and (iii) biogenesis and regulatory factors that govern Fe-S cluster assembly and distribution 26. Copper-induced stress may differentially affect these functional modules, thereby amplifying mitochondrial dysfunction through multiple routes. Disruption of metabolic Fe-S enzymes can further constrain TCA flux and exacerbate central metabolic collapse, whereas impairment of respiratory Fe-S proteins may enhance electron leakage and promote reactive oxygen species (ROS) generation. In parallel, perturbation of Fe-S biogenesis pathways may limit the replenishment of functional clusters, reinforcing a self-propagating cycle of mitochondrial dysfunction. Collectively, these processes drive the transition from initial proteotoxic injury to an irreversible bioenergetic crisis characterized by impaired respiration, elevated oxidative stress and diminished ATP production 27.

**Figure 1:**
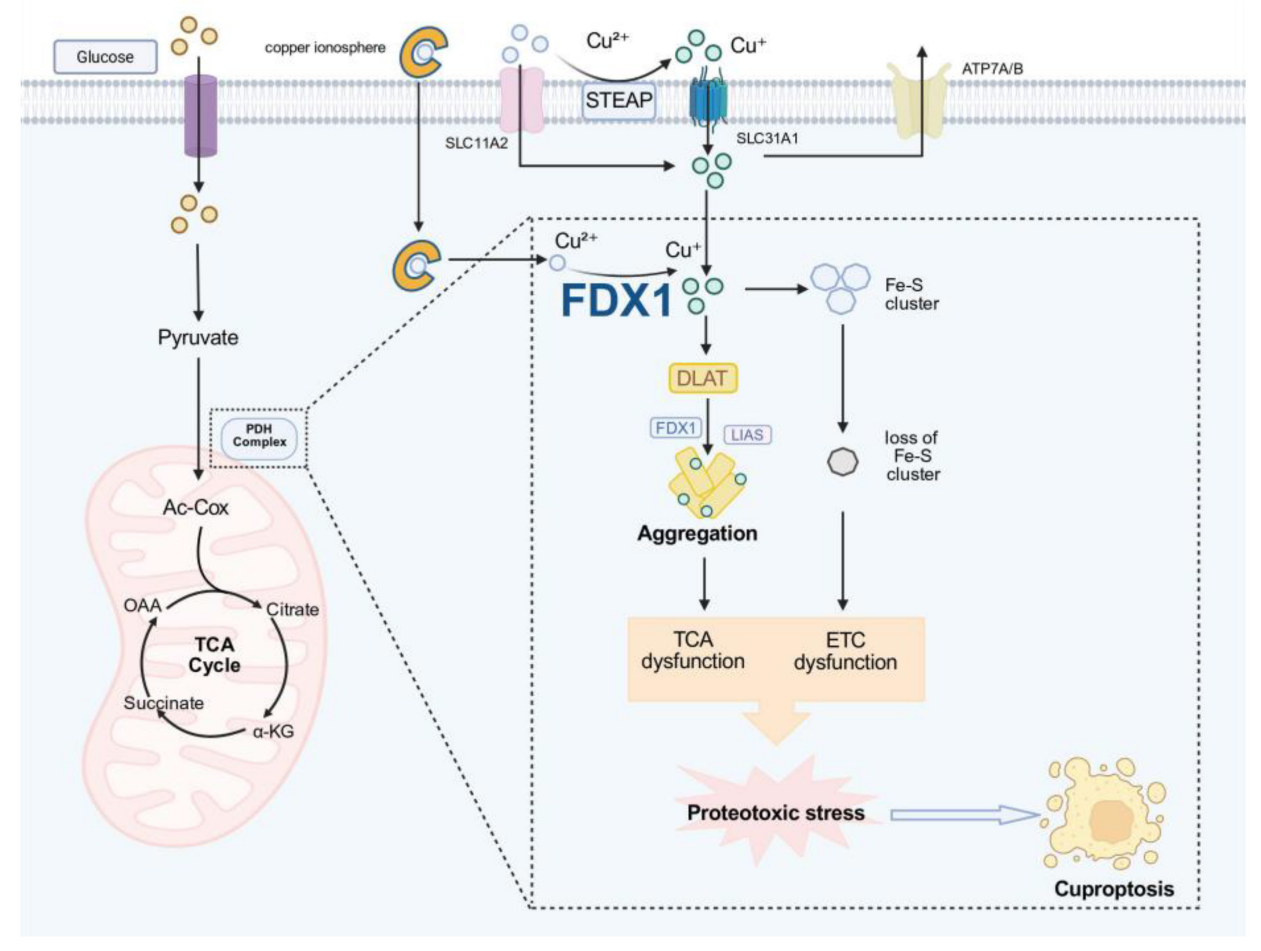
** Core regulatory mechanism of cuproptosis.** Imbalance in copper homeostasis leads to intracellular copper ion accumulation, triggering FDX1-mediated reduction of Cu²⁺ to Cu⁺. Excess Cu⁺ promotes the binding and aggregation of proteins such as DLAT and LIAS while simultaneously inhibiting Fe-S cluster biosynthesis and impairing its stability. These dual effects functionally impair the PDH complex, disrupt the TCA cycle and electron transport chain (ETC), induce mitochondrial metabolic dysfunction and proteotoxic stress, and ultimately drive cuproptosis.

As these multi-layered defects accumulate, cuproptosis proceeds toward execution, marked by TCA-cycle arrest, collapse of electron transport, dissipation of mitochondrial membrane potential and severe matrix proteotoxicity 7 (Fig. 1). This protein aggregation-driven mode of cell death is fundamentally distinct from the lipid peroxidation-based execution observed in ferroptosis, underscoring the biochemical specificity of copper-dependent cytotoxicity. Although intracellular copper buffering systems and antioxidant capacity can modulate cellular susceptibility, they do not alter the core execution machinery. Notably, cuproptosis is not governed by glutathione peroxidase 4 (GPX4)-dependent detoxification of lipid peroxides; the GPX4-glutathione (GSH) axis does not represent a primary biochemical bottleneck in this process. Nevertheless, basal GPX4-mediated redox buffering may influence overall susceptibility by mitigating concurrent oxidative stress, thereby functioning as a modulatory rather than determinative factor.

Despite its protein-centered execution mechanism, cuproptosis does not occur in isolation. The Fe-S cluster perturbation, redox imbalance and mitochondrial overload observed during copper stress partially overlap with biochemical features associated with iron-dependent oxidative injury. These observations suggest that Fe-S cluster proteins may serve as potential points of mechanistic intersection across metal-dependent cell death pathways, linking copper-induced proteotoxic stress with iron-driven oxidative damage under specific metabolic contexts 28-31. This potential connection provides a conceptual entry point for understanding ferroptosis, a lipid peroxidation-driven, iron-dependent form of cell death that shares upstream metabolic and redox determinants with cuproptosis 32.

### Mechanism of Ferroptosis

Ferroptosis is an iron-dependent form of regulated cell death that arises from a coordinated imbalance between iron-driven oxidative pressure and the intrinsic susceptibility of membrane lipids to peroxidation 6. At the molecular level, labile Fe²⁺ catalyzes Fenton reactions that continuously generate highly reactive hydroxyl radicals, thereby establishing a pro-oxidant intracellular environment. Concurrently, phospholipids enriched with polyunsaturated fatty acids (polyunsaturated fatty acid-phospholipids, PUFA-PLs) serve as the primary substrates for lipid peroxidation, providing the essential biochemical foundation for oxidative damage.

Importantly, the initiation and progression of ferroptosis are governed by two functionally distinct yet coordinated metabolic modules. First, the bioenergetic metabolism module, centered on the TCA cycle and the electron transport chain (ETC), primarily determines the intracellular ROS burden. Elevated TCA cycle flux and active oxidative phosphorylation enhance electron leakage, thereby promoting ROS accumulation and amplifying iron-mediated oxidative reactions 5, 33, 34. Second, the lipid remodeling module, exemplified by ACSL4 and LPCAT3, regulates the incorporation of PUFA into membrane phospholipids, thereby defining the susceptibility of cellular membranes to oxidative damage 35. By enriching membranes with peroxidation-prone lipid species, this module establishes the structural basis for the propagation of lipid peroxidation.

Through the coordinated action of these two metabolic layers, the core execution mechanism of ferroptosis is characterized by the uncontrolled amplification of lipid peroxidation. This process is tightly constrained under physiological conditions by cellular antioxidant systems. Among these, the GPX4-GSH axis represents the principal defense mechanism, detoxifying lipid hydroperoxides by reducing them to non-toxic lipid alcohols and thereby preserving membrane integrity 36. Disruption of this axis—whether through impaired cystine uptake, depletion of intracellular GSH, or loss of GPX4 activity—results in the collapse of antioxidant capacity, leading to the accumulation of lipid peroxides that rapidly propagate via radical chain reactions across membrane bilayers 33, 37, 38.

**Figure 2:**
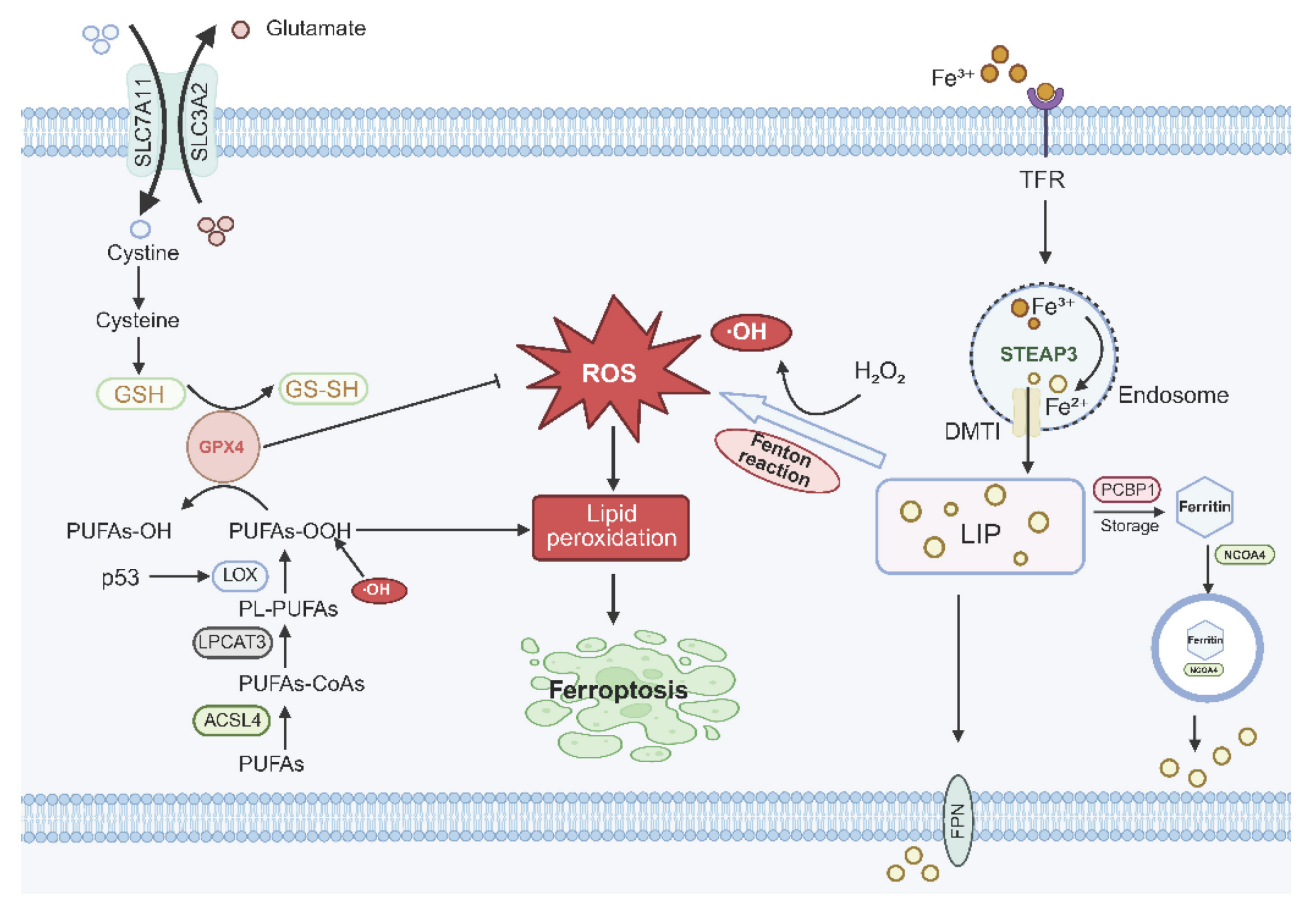
** Core regulatory mechanism of ferroptosis.** Disruption of iron homeostasis leads to intracellular accumulation of excessive Fe²+, which undergoes the Fenton reaction with H₂O₂ to generate hydroxyl radicals (·OH). These ·OH radicals deplete GPX4/GSH, impairing the antioxidant axis, and directly catalyze the peroxidation of membrane phospholipid-bound polyunsaturated-fatty acids (PL-PUFAs) into PUFA hydroperoxides (PUFAs-OOH). The persistent accumulation of lipid peroxides compromises membrane structural integrity, ultimately inducing ferroptosis.

Ferroptotic execution ensues when lipid peroxidation exceeds the threshold of membrane repair capacity. The accumulation of peroxidized phospholipids compromises bilayer integrity, disrupts ion homeostasis, and ultimately leads to catastrophic membrane failure, producing the characteristic necrotic-like morphology associated with ferroptotic cell death 39 (Fig. 2). Notably, mitochondrial metabolic status plays a critical role in modulating this threshold by regulating ROS generation capacity, whereas lipid remodeling determines membrane vulnerability to oxidative damage. Together, these two processes constitute the metabolic foundation underlying ferroptosis.

Although ferroptosis possesses a unique lipid-centered execution program, its initiation does not occur in isolation. Key biochemical features—including iron-dependent oxidative stress, instability of Fe-S cluster proteins, mitochondrial burden, and compromised antioxidant buffering—substantially overlap with those observed in copper-dependent cell death 27, 40. These shared characteristics suggest that ferroptosis and cuproptosis may be integrated within a higher-order “metal-metabolism-redox” vulnerability network, in which distinct metal ions converge on common metabolic and redox regulatory axes to determine cell fate 41.

### Shared Biological Features and the Metal-Metabolism-Redox Vulnerability Axis

Although cuproptosis and ferroptosis are executed through fundamentally distinct biochemical processes—proteotoxic collapse and lipid peroxidation, respectively—their initiation is not independent 42. Instead, both modalities arise from a shared and interconnected upstream biochemical landscape characterized by metal dyshomeostasis, increased mitochondrial metabolic burden, and compromised redox buffering capacity. Importantly, these common features should not be interpreted as generic stress conditions; rather, they operate through specific molecular nodes and dynamic interactions that channel metal-induced stress toward distinct cell death execution programs. Elucidating these coupling mechanisms is therefore essential for understanding metal-dependent cell death within a unified conceptual framework.

#### Fe-S cluster proteins as heterogeneous coupling nodes

Fe-S cluster proteins have long been regarded as central integrators linking metal homeostasis, metabolism, and redox regulation. However, conceptualizing the Fe-S system as a uniform entity obscures its substantial functional heterogeneity 43. In practice, the cellular Fe-S proteome can be broadly categorized into three functional classes: (i) metabolic Fe-S enzymes 44, 45, such as aconitase (ACO2) and lipoic acid synthetase (LIAS), which directly participate in central metabolic pathways; (ii) respiratory Fe-S proteins embedded within mitochondrial electron transport chain complexes, where they support electron transfer and energy conversion; and (iii) Fe-S biogenesis and regulatory factors, including FDX1 and ISCU, which are responsible for cluster assembly, maintenance, and distribution.

Under different metal stress conditions, these functional modules exhibit distinct patterns of vulnerability. In the context of cuproptosis, excess Cu⁺ has been associated with impaired structural integrity and functional disruption of Fe-S related proteins, particularly those involved in cluster maintenance and protein lipoylation processes. Such perturbations are thought to contribute to defective lipoylation and subsequent proteotoxic stress 33, although the precise molecular sequence remains to be fully elucidated. By contrast, ferroptosis is more directly linked to oxidative damage of Fe-S clusters, especially in metabolically active enzymes such as aconitase. Oxidative inactivation of these clusters not only disrupts TCA cycle flux but may also increase the local availability of labile iron 36, thereby reinforcing Fenton chemistry and promoting lipid peroxidation. These observations collectively indicate that Fe-S clusters function not as a single convergence node, but as a heterogeneous network of coupling elements whose differential perturbation influences cell death outcomes.

#### Candidate molecular intersections between cuproptosis and ferroptosis

Building upon Fe-S cluster perturbation as a central axis, several candidate molecular intersections may mediate the interplay between cuproptosis and ferroptosis. Although a comprehensive mapping of these interactions remains incomplete, existing evidence supports multiple layers of potential crosstalk.

First, Fe-S cluster homeostasis is tightly coupled to mitochondrial metabolism and ROS generation. Disruption of Fe-S dependent enzymes can alter both TCA cycle activity and electron transport efficiency, thereby modulating mitochondrial ROS output. This relationship is well established in ferroptosis, whereas in cuproptosis it is likely to arise as a secondary consequence of proteotoxic stress and metabolic disruption 46, 47.

Second, reciprocal interactions may exist between mitochondrial dysfunction and lipid peroxidation. In ferroptosis, mitochondrial ROS contribute to the initiation and propagation of lipid peroxidation, while reactive lipid electrophiles generated during this process have the potential to modify mitochondrial proteins and exacerbate mitochondrial stress 48, 49. Such feedback interactions may lower the threshold for proteotoxic collapse under copper stress, although direct mechanistic validation remains limited 50, 51.

Third, the GSH and thiol-redox network constitutes a shared regulatory layer. Copper accumulation can reduce intracellular thiol availability through direct binding interactions, thereby constraining glutathione-dependent antioxidant systems. As a consequence, the activity of GPX4 in detoxifying lipid peroxides may be indirectly compromised, increasing cellular susceptibility to ferroptotic triggers 52, 53.

Fourth, adaptive regulation of metal transport and redox pathways may couple copper and iron homeostasis. Stress-induced changes in cystine transport systems (such as system Xc⁻) or in metal transporter expression can simultaneously influence intracellular thiol pools and metal availability, thereby modulating sensitivity to both ferroptotic and cuproptotic stimuli 33, 54, 55. At present, these connections are supported primarily by indirect or context-dependent observations and require further mechanistic clarification 56.

#### The metal-metabolism-redox vulnerability axis

**Figure 3:**
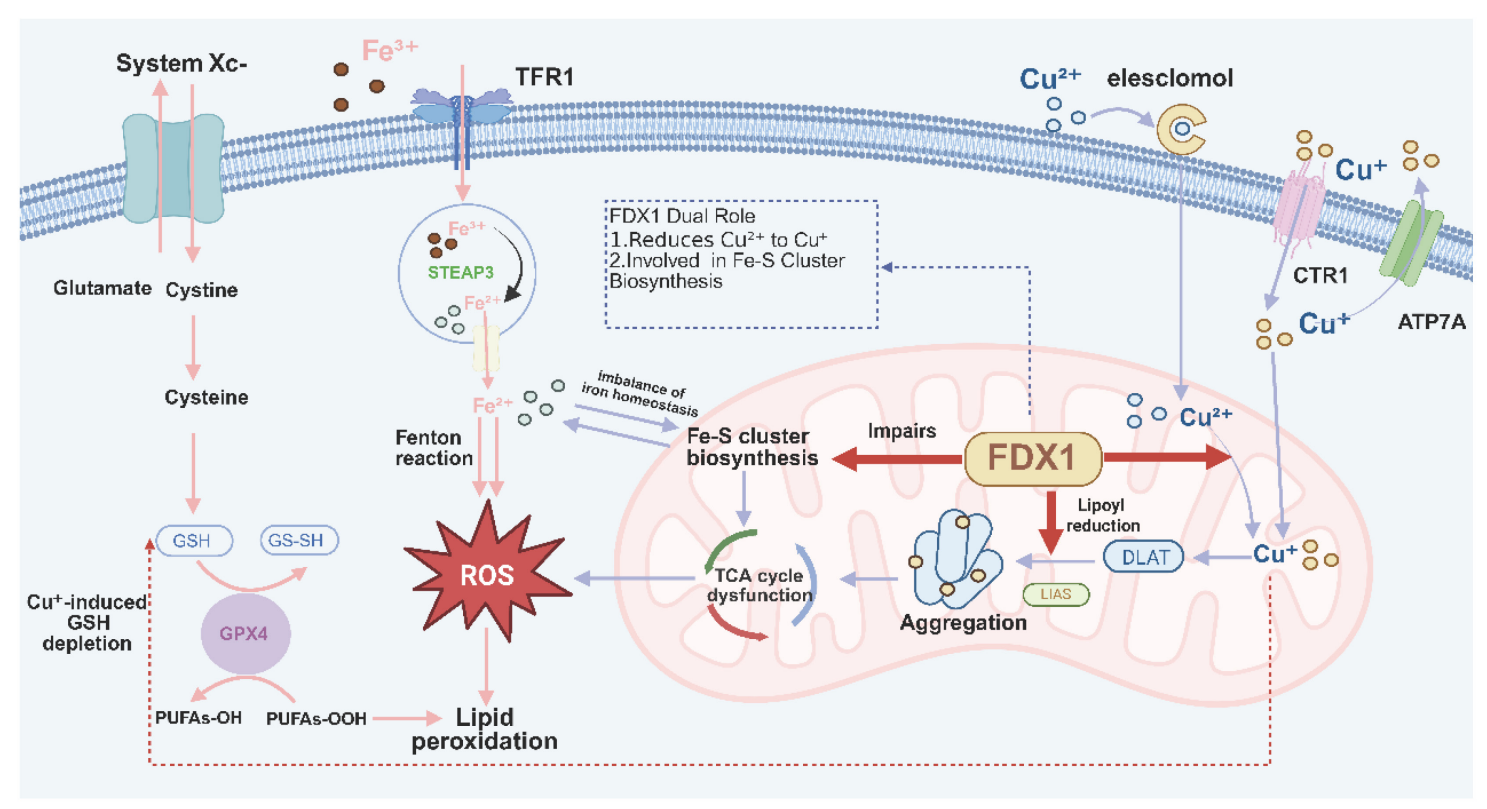
** The metal-metabolism-redox vulnerability axis linking cuproptosis and ferroptosis.** The metal-metabolism-redox vulnerability axis is defined as an integrated framework connecting copper- and iron-dependent cell death through coupled metal, metabolic, and redox processes. Metal availability is represented by copper and iron transport and accumulation, while mitochondrial metabolism is highlighted by FDX1-mediated Cu²⁺ reduction, lipoylation-dependent protein aggregation, and Fe-S cluster perturbation. Redox balance is reflected by reactive oxygen species (ROS) generation, glutathione (GSH) metabolism, and GPX4-dependent detoxification of lipid peroxides. These interdependent processes form a shared vulnerability landscape in which perturbations propagate across layers. Within this axis, copper-induced disruption of mitochondrial proteostasis promotes protein aggregation and drives cuproptosis, whereas iron-dependent ROS accumulation and lipid peroxidation favor ferroptosis. ROS serves as a central coupling signal linking mitochondrial dysfunction to membrane lipid damage, while FDX1 and GPX4 represent key regulatory nodes that bias cellular responses toward distinct execution outcomes. Thus, cuproptosis and ferroptosis emerge as context-dependent execution branches arising from a common metal-metabolism-redox constraint system rather than independent pathways.

Taken together, these heterogeneous coupling nodes and multi-layered molecular interactions support a higher-order conceptual framework, herein referred to as the metal-metabolism-redox vulnerability axis 27, 36, 57-59 (Fig. 3). This framework integrates three interdependent dimensions: intracellular metal availability, mitochondrial metabolic load, and the capacity of antioxidant defense systems 41. The interplay among these factors collectively determines cellular tolerance to metal-induced stress and defines the threshold at which adaptive responses transition into irreversible damage 33, 60, 61.

Within this axis, different forms of metal-dependent cell death can be interpreted as distinct execution outputs arising from a shared vulnerability landscape. When mitochondrial proteostasis—particularly within the matrix—is preferentially compromised, cells tend to undergo cuproptotic collapse characterized by protein aggregation. In contrast, when lipid peroxidation exceeds the repair capacity of membrane systems, ferroptotic death predominates. Thus, cuproptosis and ferroptosis are not isolated phenomena, but rather context-dependent execution branches emerging from common metabolic and redox constraints (a comparative summary is provided in Table 1) 62-64.

The relevance of this vulnerability axis becomes particularly evident in pathological conditions characterized by concurrent metal imbalance, elevated metabolic demand, and chronic oxidative stress, such as cancer, neurodegenerative diseases, and inflammatory disorders. In these settings, shared upstream stress circuits may lower the threshold for both copper- and iron-dependent cell death, positioning these modalities as integral components of a broader metal-dependent cell death network.

In addition, metal ions beyond copper and iron—including Zn, Mn, and Cd—may influence this vulnerability axis by modulating metal transport, Fe-S cluster biogenesis, and mitochondrial function, thereby further shaping context-dependent cellular outcomes.

#### Evidence hierarchy and current limitations

Despite the explanatory power of the metal-metabolism-redox vulnerability axis, it is important to recognize that the current body of evidence supporting this axis varies substantially in strength and directness. Existing studies can be broadly categorized into three levels: direct molecular evidence involving shared biochemical processes or nodes; pathway-level associative evidence observed under specific metabolic or pathological conditions; and intervention-based evidence derived from exogenous perturbations. Notably, a considerable proportion of studies reporting concurrent induction of cuproptosis and ferroptosis rely on complex experimental systems, such as metal-based nanoparticles or multifunctional nanoplatforms. While these approaches demonstrate the feasibility of co-activating multiple death pathways, they often introduce broad and pleiotropic cellular effects—including membrane disruption, lysosomal stress, and global ROS elevation—that complicate mechanistic interpretation. As such, attributing these observations to direct endogenous crosstalk between cuproptosis and ferroptosis requires careful consideration.

Future investigations employing precise genetic and biochemical perturbations—such as targeted manipulation of Fe-S related proteins or key metabolic nodes—will be essential to validate the proposed molecular intersections and to determine the extent to which this vulnerability axis operates under physiological and disease-relevant conditions.

## Disease Relevance: Pathological Manifestations of Copper- and Iron-Dependent Cell Death

Pathological conditions profoundly amplify and reconfigure the metal-metabolism-redox vulnerability axis that underlies both cuproptosis and ferroptosis, transforming what is normally a dynamically balanced regulatory system into a persistently stressed and destabilized state. In healthy tissues, metal availability, mitochondrial metabolic output, and antioxidant buffering capacity fluctuate within adaptive ranges, enabling cells to maintain homeostasis through multilayered compensatory mechanisms and to withstand transient metabolic or oxidative perturbations 65-67. In contrast, disease environments impose sustained, dysregulated, and often self-reinforcing disturbances across all three dimensions, progressively eroding the cellular capacity to buffer metal-induced biochemical stress 68, 69. For example, chronic inflammation promotes the release of labile metal ions and enhances ROS generation 70, 71; metabolic overload or energetic exhaustion compromises mitochondrial structural and functional integrity 72; and persistent oxidative stress depletes antioxidant systems required to preserve protein homeostasis and lipid integrity 73. As these pressures accumulate, perturbations that would normally be resolved become progressively entrenched, driving systemic disequilibrium across metal handling, metabolic networks, and redox regulation, and ultimately crossing the thresholds that trigger copper- or iron-dependent cell death programs 27.

Importantly, the impact of disease states on this vulnerability axis should not be interpreted as the simple activation of discrete cell death pathways. Rather, different disease types, disease stages, and cellular subpopulations occupy heterogeneous positions along a continuous spectrum defined by this axis. Some cells adopt configurations dominated by metabolic burden and metal accumulation, corresponding to an “overload-like” state, whereas others exhibit features of mitochondrial decline and antioxidant exhaustion, consistent with a “collapse-like” configuration. More commonly, these states coexist within the same tissue or shift dynamically over the course of disease progression, reflecting both spatial heterogeneity and temporal evolution. Consequently, cuproptosis and ferroptosis may be co-activated within the same cellular context, or engaged sequentially or preferentially across distinct temporal or cellular compartments.

**Figure 4:**
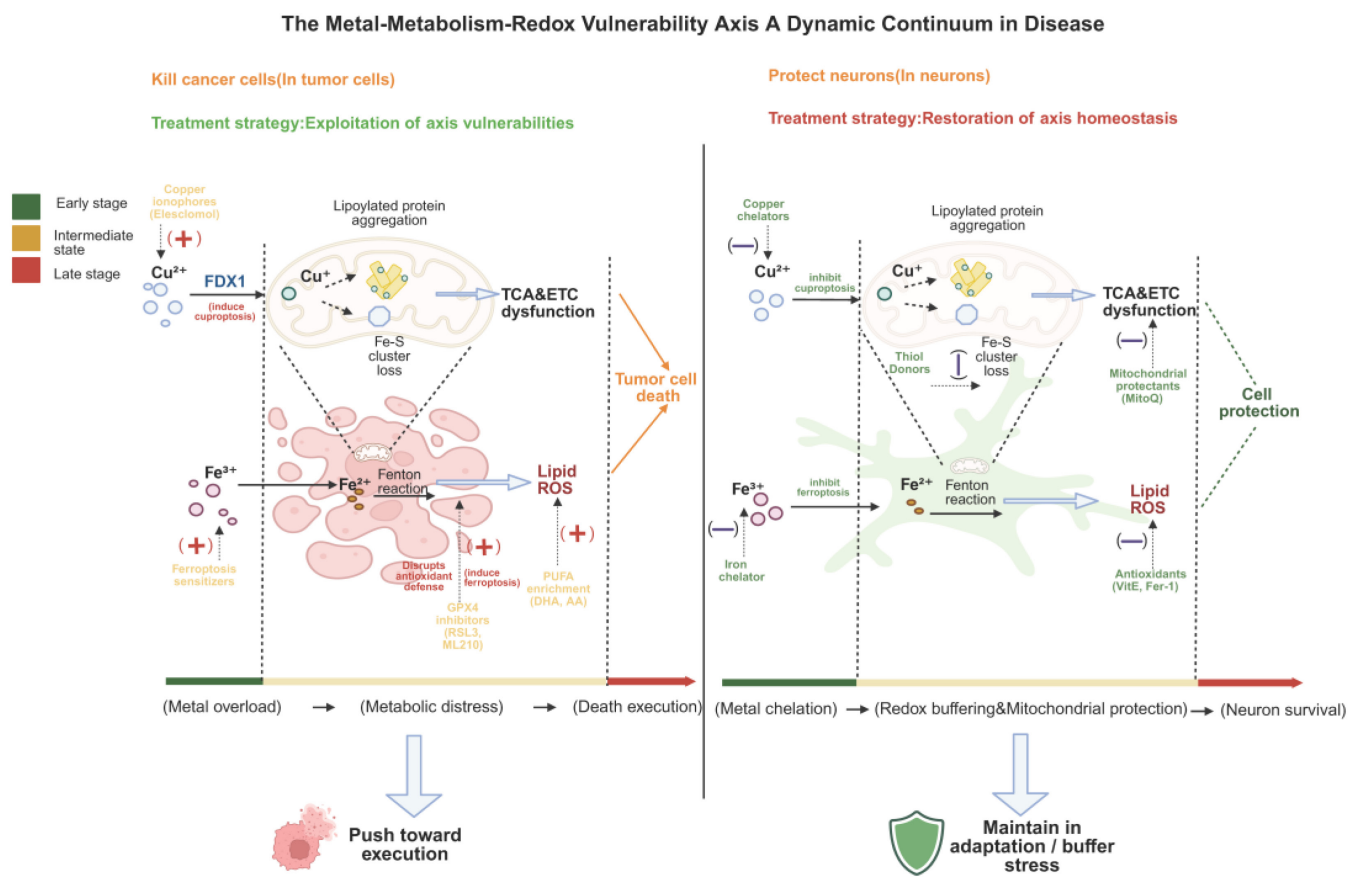
** The metal-metabolism-redox vulnerability axis as a dynamic continuum in disease.** Diseases are defined by cellular positioning along a continuous spectrum shaped by metal homeostasis, mitochondrial metabolism, and redox buffering. Representative configurations are shown for cancer and neurodegeneration, representing endpoints of a dynamic continuum rather than binary states. In cancer, elevated metal uptake, TCA/OXPHOS dependency, and PUFA-phospholipid remodeling lower thresholds for cuproptosis and ferroptosis; therapeutic strategy is to exploit the vulnerability of the axis. In neurodegeneration, chronic metal accumulation, mitochondrial dysfunction, and antioxidant depletion drive collapse; interventions focus on system stabilization via chelation, redox buffering, and mitochondrial protection. Cells shift along this axis depending on disease stage, cell type, and microenvironment, with mixed or transitional states frequently observed.

In this sense, pathological conditions do not merely activate copper- or iron-dependent cell death pathways, but instead continuously amplify and reshape the metal-metabolism-redox vulnerability axis, driving cells into a dynamic pathological state space characterized by a continuum of axis configurations. This distribution is inherently non-static and evolves under the influence of metabolic stress, therapeutic intervention, and microenvironmental changes. Within this landscape, specific axis configurations determine the relative propensity of cells to engage distinct metal-dependent death programs (Fig. 4). This perspective provides a unifying conceptual framework for understanding the recurrent emergence of cuproptotic and ferroptotic signatures across diverse diseases and offers a basis for interpreting their context-dependent heterogeneity 11, 74.

### Cancer: Axis Configurations Shaped by Metabolic Hyperactivation

A commonly observed configuration in cancer is characterized by a sustained, high-load state of the metal-metabolism-redox vulnerability axis driven by persistent metabolic and metal-associated pressures 63. Malignant cells exhibit markedly increased demands for both copper and iron to support continuous proliferation, biosynthesis, mitochondrial respiration, and epigenetic remodeling 75. Upregulation of copper transporters such as SLC31A1 76, increased transferrin receptor expression, and enhanced Fe-S cluster biosynthetic activity collectively expand intracellular labile metal pools and intensify redox-active metal cycling 77. These changes render tumor cells highly sensitive to perturbations in metal homeostasis, particularly in rapidly proliferating and metabolically active populations, where vulnerability to copper-induced proteotoxic stress and iron-driven oxidative damage is markedly increased 78.

Metabolic reprogramming further amplifies this susceptibility. Rather than relying solely on glycolysis, many tumors adopt hybrid metabolic states that combine aerobic glycolysis with elevated mitochondrial output, sustaining high TCA cycle flux while enhancing electron transport chain activity 79. This metabolic configuration increases oxygen consumption and ROS generation, continuously challenging mitochondrial proteostasis and Fe-S cluster integrity. In parallel, enhanced synthesis and remodeling of polyunsaturated fatty acid-containing phospholipids enrich cellular membranes with oxidation-prone substrates, thereby creating a permissive environment for ferroptotic lipid peroxidation 80-82. Consequently, the coordinated rewiring of metal handling and energy metabolism sensitizes tumor cells to fluctuations in intracellular copper and iron levels and significantly lowers the threshold for activation of metal-dependent cell death pathways.

Redox homeostasis is similarly placed under persistent strain. Although NRF2-driven antioxidant programs are frequently activated, sustained ROS production and intermittent metabolic surges often exceed compensatory capacity, leading to GSH depletion 83, 84, thiol oxidation, and impaired detoxification of lipid peroxides 85. Under such conditions, ferroptosis becomes an accessible execution route, particularly in tumors with pronounced ACSL4/LPCAT3-mediated PUFA-phospholipid remodeling 86. Concurrently, abnormal copper accumulation—especially in tumor subtypes with high reliance on oxidative phosphorylation (OXPHOS)—creates conditions favorable for lipoylated protein aggregation, exacerbated mitochondrial proteotoxic stress, and cuproptosis-like collapse 87-89.

Importantly, emerging evidence suggests that overload should be interpreted as a predominant configuration observed in many actively proliferating tumor cells rather than a fixed pathological identity. As disease progresses, and under conditions such as nutrient limitation, cachexia, or sustained therapeutic pressure, subsets of tumor cells may progressively acquire features more characteristic of collapse-like states, including mitochondrial dysfunction, antioxidant exhaustion, and impaired metal buffering. Thus, different stages of cancer, distinct cellular subpopulations, and even spatially separated regions within the same tumor may occupy different positions along the metal-metabolism-redox vulnerability axis, reflecting heterogeneous regulatory states of metal-dependent cell death susceptibility.

From a therapeutic perspective, this vulnerability axis indicates that effective anticancer strategies should not be limited to the indiscriminate activation of a single cell death pathway, but rather should be guided by the specific axis configurations exhibited by tumor cells. For example, in tumors characterized by elevated metal accumulation, active OXPHOS, and fragile mitochondrial proteostasis, copper ionophores, modulators of copper distribution, or strategies that enhance copper-induced stress may preferentially induce cuproptosis. In contrast, tumors with pronounced PUFA remodeling and compromised lipid peroxide detoxification may be more susceptible to ferroptosis-inducing interventions 90, 91. Therefore, the therapeutic potential of cuproptosis- and ferroptosis-targeting strategies across diverse malignancies likely arises not merely from their ability to activate specific death pathways, but from their capacity to exploit context-dependent imbalances within the metal-metabolism-redox vulnerability axis 92.

### Neurodegeneration: Dynamic Axis Configurations Under Progressive Vulnerability

In contrast to the metabolically hyperactivated states frequently observed in cancer, neurodegenerative diseases are more commonly characterized by regulatory configurations of the metal-metabolism-redox vulnerability axis that shift toward the collapse end. Importantly, such a collapse-leaning state should not be interpreted as a fixed endpoint, but rather as a dynamic outcome that evolves over the course of disease progression. During early or compensatory stages, subsets of neurons may transiently maintain homeostasis through upregulation of antioxidant defenses or localized metabolic activity, thereby exhibiting partial “overload-like” adaptive features. However, with aging and the cumulative burden of pathological stress, these compensatory mechanisms progressively deteriorate, ultimately driving cells toward a collapse-dominant state characterized by energy insufficiency, metal dyshomeostasis, and depletion of redox buffering capacity 93-95. Neurons, owing to their high dependence on oxidative phosphorylation, limited regenerative potential, and long lifespan, are particularly susceptible to this transition 93.

At the level of metal handling, disruption of metal homeostasis emerges as a critical driver of this shift. As disease progresses, aberrant accumulation and redistribution of copper and iron increasingly occur within synaptic compartments, mitochondria, and pathological protein aggregates 96. Impairment of metal chaperone systems, defective metal efflux mechanisms, and abnormal metal-protein interactions collectively exacerbate this imbalance, simultaneously increasing the risk of copper-mediated proteotoxicity and iron-driven lipid peroxidation 97. Notably, in early disease stages, these alterations may display heterogeneous distributions across neuronal subtypes or subcellular compartments, generating varying degrees of metal stress and further amplifying spatial heterogeneity within the system.

Mitochondrial dysfunction constitutes another central pathological hallmark of neurodegenerative diseases 98. Loss of electron transport chain components, reduced TCA cycle flux, and fragmentation of mitochondrial networks collectively impair ATP production while elevating local ROS levels 99, 100. Declines in Fe-S cluster biogenesis and repair further compromise the functionality of key metabolic enzymes and redox regulatory proteins, rendering mitochondria increasingly vulnerable under sustained stress conditions 101. Beyond energy failure, these alterations also disrupt the coordination between metal homeostasis and redox regulation through Fe-S dependent protein networks, thereby creating a permissive environment for both cuproptosis-associated mitochondrial proteotoxicity and ferroptosis-associated lipid peroxidation 102. Importantly, the relative contribution of these processes may vary across disease stages and neuronal subpopulations, further reflecting the dynamic distribution along the vulnerability axis.

As a downstream consequence, progressive exhaustion of redox buffering systems provides a key molecular basis for axis destabilization. Reduced GSH levels, irreversible oxidation of thiol groups, accumulation of lipid peroxides, and impairment of mitochondrial quality control collectively establish a persistent pro-oxidative environment, closely resembling the upstream conditions that trigger ferroptosis 103. In parallel, oxidative modification of mitochondrial proteins, decline in chaperone activity, and progressive failure of proteostasis systems increase neuronal susceptibility to copper-induced protein aggregation and proteotoxic stress. Molecular features associated with ferroptosis and cuproptosis frequently coexist with classical protein aggregation pathologies, including Aβ, α-synuclein, and TDP-43, suggesting that metal-dependent cell death processes may act synergistically with proteotoxic stress rather than functioning as isolated events 104-106.

These mechanistic insights also have direct implications for therapeutic intervention. In contrast to cancer therapies that aim to induce cell death, strategies in neurodegenerative diseases are primarily directed toward restoring or buffering the imbalance of the vulnerability axis. For example, metal chelators or approaches that modulate metal redistribution may alleviate toxicity associated with abnormal metal accumulation; antioxidant interventions and enhancement of GSH metabolism can partially re-establish redox balance; and mitochondrial-protective strategies, such as improving electron transport chain function or enhancing mitochondrial quality control, may increase cellular resilience to metabolic and oxidative stress. Rather than targeting a single death pathway, these interventions act by reshaping the global state of the metal-metabolism-redox vulnerability axis, thereby reducing the likelihood of neurons entering irreversible cell death programs.

Taken together, metal-dependent cell death in neurodegenerative diseases does not arise from a single molecular event, but from the progressive loss of stability within the metal-metabolism-redox vulnerability axis. During this process, the coordinated regulation among metal handling, mitochondrial metabolic resilience, and antioxidant systems is gradually compromised, driving cells toward the collapse end of the axis. However, this transition is inherently stage-dependent and heterogeneous, with different cell populations and disease stages occupying distinct positions along the axis, resulting in diverse susceptibilities to metal-dependent cell death 107, 108. Importantly, this dynamic distribution not only captures the evolving nature of neurodegenerative pathology, but also provides a conceptual lens for a more integrative understanding of its connections with other disease contexts, including cancer.

### Cross-Disease Axis Configurations: A Dynamic Continuum of Vulnerability

Across diverse pathological contexts, cancer and neurodegenerative diseases exhibit distinct yet interconnected configurations of the metal-metabolism-redox vulnerability axis 40, 41. Rather than constituting discrete or opposing categories, these configurations are more appropriately understood as dynamic distributions along a shared regulatory continuum. Cellular states are not confined to fixed modes; instead, they continuously reposition along this axis in response to disease progression, microenvironmental cues, and intrinsic regulatory constraints. In this context, so-called “hyperactivated” or “collapse-leaning” states are better interpreted as stage-dependent or subpopulation-specific features, rather than stable and defining properties 109.

From a temporal perspective, axis configurations may vary substantially throughout disease progression. For instance, during early tumor development, cells often display elevated metabolic activity and increased metal demand 110. However, under therapeutic pressure or nutrient limitation, subsets of tumor cells may shift toward states characterized by energy restriction and diminished redox buffering capacity, reflecting a transition toward heightened vulnerability. A similar dynamic can be observed in neurodegenerative diseases, where certain neurons in early stages may transiently maintain functional stability through enhanced metabolic activity or activation of antioxidant responses, thereby exhibiting adaptive regulatory features 68. These observations indicate that different disease types do not simply occupy opposite ends of the axis, but instead undergo progressive transitions from relatively balanced states to increasingly destabilized configurations over time.

In addition to temporal dynamics, spatial and cellular heterogeneity further modulate the positioning of cells along the vulnerability axis. Even within a single tissue or pathological context, distinct cell types, subcellular compartments, and microenvironmental niches may experience varying degrees of metal stress, metabolic burden, and redox imbalance. Such heterogeneity not only influences cellular susceptibility to metal-dependent cell death, but also shapes the spatial distribution and interplay of distinct death mechanisms within tissues 111.

At the mechanistic level, this cross-disease dynamic distribution reflects a unified regulatory logic in which metal homeostasis, mitochondrial metabolism, and redox control are tightly interconnected rather than independently operating processes. Within this integrated system, cuproptosis and ferroptosis are more appropriately conceptualized not as isolated pathways, but as distinct execution modes that are preferentially engaged under specific axis configurations. For example, conditions dominated by mitochondrial metabolic stress and proteotoxic burden may favor cuproptosis-associated responses characterized by mitochondrial protein aggregation, whereas states driven by lipid peroxidation and depletion of antioxidant defenses are more likely to trigger ferroptosis-associated membrane damage. These differences in execution primarily arise from variations in upstream regulatory states, rather than fundamentally separate molecular programs 38, 112.

Importantly, this regulatory logic is not restricted to cancer and neurodegenerative disorders. Similar patterns of metal imbalance, mitochondrial stress, and redox dysregulation are observed in ischemia-reperfusion injury, metabolic disorders, and chronic inflammatory conditions, often accompanied by features indicative of ferroptotic or cuproptotic engagement. This suggests that metal-dependent cell death represents a convergent outcome under diverse pathological pressures, rather than a phenomenon confined to specific disease categories.

Overall, cellular states do not transition between entirely independent death pathways; instead, they dynamically distribute and evolve within a continuous regulatory space defined by the “metal-metabolism-redox vulnerability axis”. Within this space, cuproptosis and ferroptosis function as context-dependent execution modes that jointly contribute to cell fate determination. This continuum-based perspective facilitates the integration of molecular mechanisms across different diseases and provides a refined conceptual basis for understanding the regulation of metal-dependent cell death 4. To further consolidate this axis-based perspective, representative configurations across different pathological contexts, together with their associated functional implications and execution tendencies, are summarized in Table 2.

The metal-metabolism-redox vulnerability axis defines a continuous regulatory space across diverse disease contexts. Cellular configurations dynamically shift along this axis in a stage-, cell type-, and microenvironment-dependent manner. Core dimensions—including metal homeostasis, mitochondrial function, Fe-S cluster integrity, and redox buffering—jointly determine cellular positioning within this continuum. Within this context, cuproptosis and ferroptosis are best understood as execution modes engaged under distinct axis configurations, rather than as isolated pathways.

## A Unified Metallo-Redox-Metabolic Framework

### Conceptual Rationale and Structural Organization of the Unified Framework

**Figure 5:**
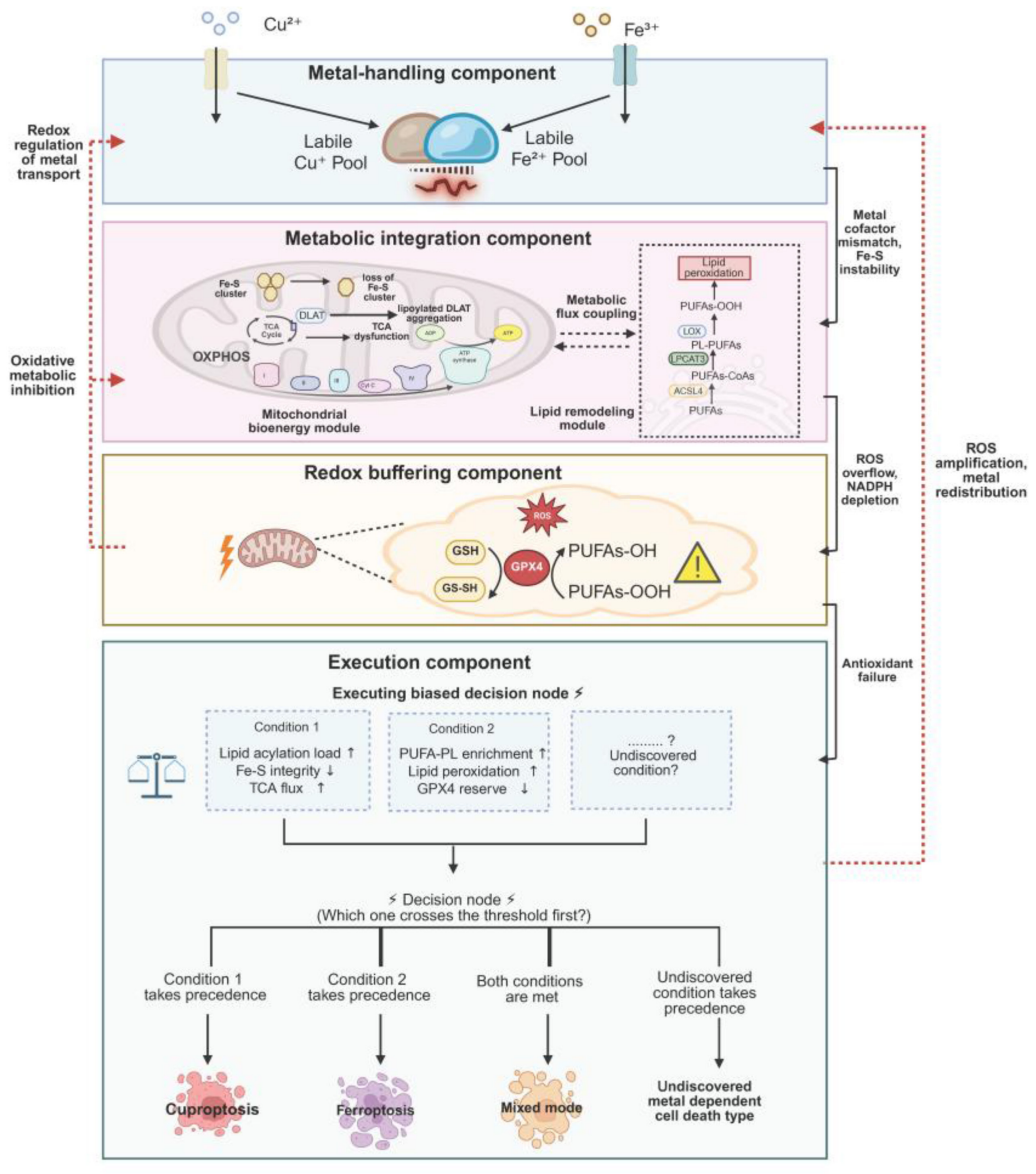
** A dynamic decision model of the unified metallo-redox-metabolic framework.** The framework organizes cellular responses into four interconnected components. Bidirectional arrows indicate inter-layer communication and feedback regulation; solid arrows represent forward propagation, and dashed arrows represent feedback regulation. Metal-handling component: Labile Cu⁺/Fe²⁺ pools establish upstream stress. Metabolic integration component: Subdivided into mitochondrial bioenergetic module (TCA cycle, OXPHOS, Fe-S integrity, lipoylated enzyme burden) and lipid remodeling module (ACSL4/LPCAT3, PUFA-PL enrichment), which couple to shape stress amplification. Redox buffering component: GSH-GPX4 axis maintains redox balance, with bidirectional feedback to metal handling and metabolic states (oxidative inhibition; redox-regulated transport). Execution component: A decision node integrates competing inputs. The framework predicts threshold-dependent switching: cuproptosis is favored when mitochondrial lipoylation and Fe-S burden reach a critical level prior to GPX4 depletion, whereas ferroptosis is favored when PUFA remodeling and lipid peroxidation exceed GPX4 buffering capacity; mixed states may arise when both thresholds are approached. All predictive variables are experimentally measurable: lipoylation by western blot, Fe-S integrity by aconitase activity, GPX4 reserve by activity assay, lipid peroxidation by C11-BODIPY.

The previously described metal-metabolism-redox vulnerability axis indicates that cellular susceptibility to metal-dependent cell death arises from the coordinated influence of multiple intrinsic states, including metal homeostasis, mitochondrial metabolic load, and redox regulatory capacity. These factors vary continuously across different cell types and pathological contexts, collectively shaping the overall vulnerability of cells under stress conditions. Within this continuum, cuproptosis and ferroptosis can be understood as distinct terminal manifestations that emerge under specific configurations of this state space. To further elucidate how these cellular states are sensed, integrated, and ultimately translated into divergent cell death outcomes, we propose a unified metal-redox-metabolism framework grounded in the concept of a biological vulnerability axis (Fig. 5). This framework is designed to describe the hierarchical organization and dynamic propagation of metal-associated stress within cells 113, 114. Accumulating evidence suggests that, prior to the onset of both cuproptosis and ferroptosis, cells undergo a series of convergent alterations, including fluctuations in metal availability 36, increased mitochondrial metabolic pressure, disruption of Fe-S cluster-associated functions, and progressive weakening of redox buffering capacity. These observations indicate that metal-dependent cell death does not arise from a single signaling pathway, but rather from the coordinated imbalance of multiple regulatory dimensions.

Within this framework, metal-associated stress can be organized into four functionally interconnected components: metal handling, metabolic integration, redox buffering, and execution. These components operate as a tightly coupled system, linked through continuous signal propagation and feedback regulation, thereby progressively transforming initial metal perturbations into irreversible cellular damage (Table 3).

The upstream metal-handling component encompasses the uptake, transport, storage, and compartmentalization of copper and iron, determining the dynamic availability of intracellular labile Cu⁺ and Fe²⁺ pools 115, 116. Fluctuations in metal ion concentrations provide the primary stress input to the system and are further propagated downstream by influencing metal cofactor binding, mitochondrial metal loading, and Fe-S cluster-related processes. Through these mechanisms, copper and iron jointly shape the intracellular metal environment and establish the initial conditions for subsequent stress responses 44, 117, 118.

The metabolic integration component serves as a central hub that links metal perturbations to cellular damage by integrating and amplifying stress signals. Functionally, this component can be divided into two interconnected modules. The first is the mitochondrial bioenergetic module, encompassing the TCA cycle, oxidative phosphorylation, and Fe-S cluster biogenesis and maintenance 73. This module governs cellular energy metabolism, electron transport efficiency, and ROS production under metal stress. The second is the lipid remodeling module, which involves the synthesis of polyunsaturated phospholipids and the dynamic regulation of membrane architecture, thereby influencing the susceptibility of cellular membranes to oxidative damage. Together, these modules shape cellular response patterns by modulating both mitochondrial stress and membrane vulnerability. In addition, metabolic alterations further influence redox homeostasis through increased ROS generation, NADPH consumption, and elevated thiol demand 119-122.

The redox buffering component plays both integrative and regulatory roles within this framework. Centered on the GSH-GPX4 system and supported by broader thiol-dependent networks, this component maintains intracellular redox balance. Under manageable stress conditions, it preserves system stability by eliminating reactive oxygen species and lipid peroxides. As stress intensifies, however, redox buffering capacity progressively declines and, in turn, modulates metal handling and metabolic activity 123, 124. For instance, changes in the redox environment can alter the functional states of metal transporters and affect the activity of Fe-S cluster associated enzymes as well as mitochondrial metabolic enzymes, thereby reshaping the overall metabolic configuration of the cell. Once buffering capacity approaches exhaustion, cells gradually lose control over oxidative damage and transition into an irreversible injury state 125.

Under the combined influence of these interconnected processes, cells ultimately enter the execution component, where distinct forms of metal-dependent cell death become manifest. At this stage, different vulnerable cellular structures may undergo destabilization. For example, when mitochondrial lipoylated protein stress, TCA cycle burden, and Fe-S cluster dysfunction reach critical levels, cells tend to exhibit a cuproptosis-like phenotype characterized by mitochondrial proteotoxicity and metabolic collapse 126. Conversely, when lipid peroxidation within cellular membranes exceeds the regulatory capacity of antioxidant systems such as GPX4, ferroptotic phenotypes dominated by membrane damage are more likely to occur 127, 128. In certain contexts, these damage processes may coexist, giving rise to hybrid execution patterns. Thus, the emergence of specific cell death modalities reflects the cumulative effects of multilayered stress across distinct structural and functional units, rather than the consequence of a single determinant.

Collectively, the unified framework integrates metal homeostasis, metabolic configuration, and redox regulation into a continuous functional system. It provides a structural basis for understanding the context-dependent manifestations of metal-dependent cell death across different cell types and disease conditions, and offers a conceptual foundation for interpreting the dynamic interplay and regulatory potential underlying cuproptosis and ferroptosis 69.

### Dynamic Behavior, Crosstalk, and Operating Constraints of the Unified Framework

Within the unified metallo-redox-metabolic framework, cellular responses to metal-associated stress do not occur instantaneously but instead exhibit a continuous and dynamic progression. As stress accumulates, cells typically transition through a series of states, ranging from adaptation to vulnerability and subsequently to irreversible damage. During the initial phase, homeostasis is maintained through coordinated regulation of metal transport, metabolic flux, and antioxidant systems. With sustained stress, the system gradually shifts toward a vulnerable state, in which metabolic pressure, oxidative burden, and metal imbalance begin to reinforce one another. As key buffering capacities approach their limits, cells enter a commitment phase characterized by a marked decline in the ability to control damage, ultimately progressing to an irreversible execution state. This progression reflects a gradual shift of cellular states within a continuous landscape, rather than an abrupt, discrete transition.

In addition to temporal dynamics, metal-associated stress also exhibits pronounced spatial heterogeneity within cells. Distinct subcellular compartments assume different functional roles under stress conditions and display differential susceptibility to damage. Mitochondria, as central hubs of energy metabolism and Fe-S cluster biogenesis, are particularly prone to accumulating proteotoxic stress and metabolic imbalance under elevated metal load and metabolic strain. In contrast, cellular membranes, especially those enriched in polyunsaturated phospholipids, display heightened sensitivity to oxidative damage during lipid peroxidation processes. As a result, different structural units within the cell may reach their respective damage thresholds at distinct time scales, giving rise to divergent execution trajectories. This spatial heterogeneity contributes to the phenotypic diversity observed in metal-dependent cell death.

Throughout this dynamic progression, functional components of the framework interact through multiple forms of coupling that collectively shape system behavior. Metal handling, metabolic integration, and redox buffering are not organized in a simple linear cascade but instead form a tightly interconnected network regulated by resource allocation, metabolic flux, and redox state. Fluctuations in metal ion availability not only directly influence cofactor-dependent enzymatic activity but also indirectly modulate mitochondrial function and metabolic flux, thereby affecting the generation and clearance of ROS. Conversely, changes in the redox environment can alter the functional states of metal transporters and the activity of metabolic enzymes, establishing multi-layered feedback regulation. Under specific conditions, different stress modalities may exhibit competitive or biased interactions, thereby influencing the dominant direction of instability within the system. When mitochondrial-associated stress predominates, execution pathways characterized by metabolic collapse are more likely to emerge, whereas dominance of lipid peroxidation favors membrane-centered execution modes 129, 130.

The operation of this framework is further constrained by specific biological conditions. Functional mitochondrial networks and active metabolic processes are essential for integrating metal-derived stress, particularly in relation to energy metabolism and Fe-S cluster associated pathways. In parallel, the capacity for lipid metabolism and membrane remodeling determines cellular susceptibility to oxidative damage. An intact redox buffering system is equally critical for maintaining reversibility and delaying the progression toward irreversible injury. In the absence of these key functional elements, the influence of the metallo-redox-metabolic axis on cell fate determination is substantially diminished, and the overall system behavior is correspondingly altered.

Under the combined influence of dynamic progression and these operating constraints, the unified framework provides not only a coherent explanation for distinct forms of metal-dependent cell death but also a basis for their potential prediction. The position of a cell along the vulnerability axis, the spatial distribution of stress across subcellular compartments, and the coupling states among regulatory components together influence the likelihood of adopting specific execution trajectories. In this context, metal-dependent cell death is more appropriately understood as the outcome of continuous state evolution within a multidimensional regulatory network, rather than as isolated or discrete processes 131.

### Systematic Reframing of Metal-Dependent Cell Death and Disease Mechanisms within a Unified Framework

In previous studies, the mechanisms underlying cuproptosis and ferroptosis have largely been elucidated through the progressive characterization of key molecules, signaling pathways, and specific biomarkers. These efforts have provided important insights into the roles of metal ions in regulating cell death and have led to substantial mechanistic understanding across diverse pathological contexts, including cancer and neurodegenerative diseases. However, from a global perspective, most of these studies remain centered on individual pathways or isolated molecular events. The intrinsic connections among different findings, as well as their positions within higher-order regulatory networks, are often unclear, resulting in a fragmented overall landscape. Such a reductionist paradigm, while informative at the local level, limits a deeper understanding of the systemic regulatory logic governing metal-dependent cell death.

The unified metal-redox-metabolic framework provides a new integrative perspective to address this limitation. By incorporating metal homeostasis, metabolic state, and redox regulation into a single analytical system, this framework establishes a continuous regulatory space in which previously dispersed findings can be mapped onto a coherent structural and functional hierarchy. Within this context, individual molecular events are no longer interpreted in isolation but are understood as functional manifestations of distinct components within the system. Their roles must therefore be evaluated in relation to their hierarchical position and their coupling with other regulatory modules. Consequently, this framework not only offers an organizing principle for existing knowledge but also provides a systematic basis for uncovering intrinsic connections and coordinated interactions among different mechanisms.

From this perspective, cuproptosis and ferroptosis can be reinterpreted as distinct execution outcomes of a shared stress system under different system configurations. Cuproptosis can be conceptualized as the localized accumulation and amplification of multi-layered stress within mitochondria-associated structures. At the level of metal handling, copper uptake and accumulation initiate the primary perturbation. In the metabolic integration component, interactions involving lipoylated proteins, alterations in TCA cycle flux, and disruption of Fe-S cluster integrity further intensify mitochondrial stress. Meanwhile, a decline in redox buffering capacity weakens the system's ability to manage proteotoxic and metabolic imbalances. When these perturbations spatially converge on mitochondria and exceed the system's tolerance threshold, the execution layer manifests a cuproptotic phenotype characterized by proteotoxic stress and metabolic collapse.

In contrast, ferroptosis reflects the preferential accumulation and destabilization of stress within membrane structures. Iron-dependent oxidative reactions, lipid metabolic processes that shape membrane susceptibility, and the capacity of antioxidant systems to counteract lipid peroxidation collectively determine the extent of stress accumulation at the membrane level. When redox buffering becomes insufficient to maintain lipid homeostasis, the system transitions into a ferroptotic state dominated by membrane damage at the execution stage. Thus, these two forms of cell death can be understood as distinct execution pathways arising from shared upstream perturbations but diverging in the structural units where instability ultimately occurs.

Importantly, the unified framework enables a deeper understanding of the relationship between cuproptosis and ferroptosis. At the upstream regulatory level, both processes are driven by common factors, including disturbances in metal homeostasis, metabolic stress, and redox imbalance. However, at the execution stage, they diverge in their dominant substrates and damage modalities, corresponding to preferential destabilization of mitochondria-associated versus membrane structures. Under certain conditions, multi-layered stress may accumulate simultaneously across multiple structural units, giving rise to hybrid execution patterns with overlapping features. In this sense, cuproptosis and ferroptosis should not be viewed as independent cell death pathways but rather as two manifestations of a shared regulatory system following a “common origin-divergent execution” paradigm. This structural perspective not only clarifies the relationships and potential transitions between different death modalities but also provides a unified theoretical basis for understanding their cooperation and competition in complex biological contexts.

More importantly, this framework offers a systematic strategy for integrating disease related findings. In traditional studies, disease mechanisms are often investigated in a molecule- or pathway-centric manner. While such approaches reveal critical local mechanisms, the positioning of these findings within the broader regulatory network and their connections to other processes remain poorly defined. By mapping these seemingly independent observations onto distinct functional layers and regulatory modules within the unified framework, cross-study integration and mechanistic linkage can be achieved 61, 125.

For instance, in cancer research, mechanisms involving SLC7A11-mediated cystine uptake and glutathione synthesis, GPX4-dependent detoxification of lipid peroxides, and ACSL4-driven polyunsaturated fatty acid metabolism are often studied separately from the perspectives of redox regulation or lipid metabolism. Within the unified framework, these processes can be mapped to the redox buffering and metabolic integration component, respectively, and further linked through their coupling with metal-dependent oxidative reactions. Together, they define the extent of lipid peroxidation accumulation and the threshold for ferroptotic execution. Through this integrative view, these seemingly independent molecular processes are reorganized into a continuous regulatory chain, providing a more coherent explanation for the variability in ferroptosis sensitivity observed in tumor cells under different metabolic and redox states.

Similarly, in neurodegenerative diseases, alterations in copper transport (e.g., ATP7A/ATP7B), mitochondrial dysfunction, and the accumulation of oxidative stress are typically studied within separate domains, such as metal metabolism, energy metabolism, or oxidative damage. Within the unified framework, these alterations can be assigned to dysfunctions in the metal-handling, metabolic integration, and redox buffering components, respectively. Their coordinated effects at the system level progressively drive neurons toward a “low-buffering, high-vulnerability” state configuration. This cross-level integration not only helps explain the mechanisms underlying metal-associated neuronal damage but also provides a unified perspective on the chronic progression of neurodegenerative diseases and their heightened sensitivity to sustained stress.

Through this process of “mapping-integration-reframing”, the unified framework connects previously fragmented findings at the system level. It clarifies the functional positioning of diverse molecular events within the overall regulatory network and provides a clear path for exploring deeper regulatory relationships. Within this perspective, cuproptosis, ferroptosis, and their manifestations in different disease contexts are no longer interpreted through isolated pathways but are understood as the outcomes of coordinated interactions among multi-layered regulatory factors under specific system configurations. Accordingly, metal-dependent cell death can be viewed as a dynamic process evolving within a continuous state space, for which the unified framework provides a coherent and systematic explanatory foundation.

### Predictive Capacity and Testable Hypotheses within the Unified Framework

The unified metallo-redox-metabolic framework transforms metal-dependent cell death from a descriptive synthesis into a predictive systems-level model. Within this framework, cuproptosis and ferroptosis are interpreted as execution outcomes jointly determined by multi-layered system states rather than as independent pathways. This perspective enables the formulation of a series of testable, system-level predictions, providing a conceptual basis for mechanistic investigation of metal-dependent cell death.

#### Execution Bias: System State Determines Death Pathway Selection

A central prediction of the framework is the existence of execution bias. Under comparable metal stress, the likelihood of a cell undergoing cuproptosis or ferroptosis is not dictated by metal identity alone, but by the integrated state of metal homeostasis, metabolic configuration, and redox buffering capacity. In this sense, the execution mode reflects the global system state rather than the activation of a single pathway 132.

This bias can be approximated through a set of measurable system variables. For instance, mitochondrial lipoylated protein burden (e.g., the lipoylation level of DLAT) reflects copper-dependent proteotoxic stress, whereas Fe-S cluster integrity (e.g., aconitase activity) indicates the stability of key mitochondrial enzymatic systems. The degree of reliance on TCA cycle and oxidative phosphorylation further captures metabolic dependency on mitochondrial function. In parallel, lipid peroxidation levels (e.g., C11-BODIPY oxidation) and antioxidant reserve capacity (e.g., GPX4-GSH status) define the threshold for membrane oxidative damage. Importantly, these variables are not independent but are dynamically coupled, collectively shaping the distribution of stress across regulatory layers.

Within this multidimensional state space, cell death outcomes can be understood as the result of crossing distinct system-level thresholds. Specifically, when mitochondrial-associated stress—characterized by elevated lipoylated enzyme burden and TCA/Fe-S strain—reaches a critical level prior to the exhaustion of antioxidant defenses, the system preferentially shifts toward a cuproptosis-biased execution mode marked by proteotoxic collapse and metabolic disruption. Conversely, when PUFA-driven membrane remodeling and lipid peroxide propagation exceed the buffering capacity of GPX4, the system is more likely to transition toward ferroptotic membrane failure. This reflects a competition between regulatory bottlenecks across different system layers.

Importantly, this prediction is experimentally testable. For example, graded perturbations of copper or iron levels can be combined with targeted metabolic rewiring—such as enforcing glycolytic versus oxidative metabolic states or modulating PUFA availability—to assess shifts in execution bias under defined initial conditions. Real-time monitoring of lipoylation dynamics and lipid peroxidation signals can further be used to determine whether the observed death modality aligns with the pre-perturbation system configuration.

#### Threshold-Dependent Switching: Temporal Reconfiguration of Death Modalities

Beyond execution bias, the framework predicts that metal-dependent cell death exhibits pronounced dynamic plasticity. Under sustained or evolving stress conditions, cells may undergo threshold-dependent switching between distinct execution modes. This transition is driven by time-dependent changes in key system variables, including progressive depletion of GSH reserves, dynamic fluctuations in labile copper and iron pools, accumulation of mitochondrial ROS, and gradual destabilization of Fe-S clusters. As these processes evolve, the dominant limiting factor within the system may shift. For instance, cells initially biased toward a cuproptosis-like state driven by mitochondrial proteotoxic stress may progressively transition to a ferroptotic mode as antioxidant defenses become critically depleted and lipid peroxidation becomes the primary source of damage 133.

Such dynamic switching is particularly relevant in disease contexts characterized by chronic metal imbalance or sustained metabolic stress. In these settings, different cell populations—or even the same tissue over time—may exhibit distinct death phenotypes, giving rise to pronounced spatiotemporal heterogeneity.

This prediction can be evaluated using time-resolved, multi-omics approaches. For example, longitudinal profiling of transcriptomic, metabolomic, and lipidomic changes under continuous sub-lethal metal stress, combined with temporally controlled application of pathway-specific inhibitors (e.g., ferrostatin-1 or copper chelators), may help identify critical transition points at which the dominant execution modality shifts.

#### Disease-Associated Predictions: System Configuration-Driven Vulnerabilities and Intervention Windows

At the disease level, the framework further predicts that distinct pathological conditions do not simply correspond to the activation of specific cell death pathways, but rather to characteristic configurations along the metallo-redox-metabolic axis. Such system configurations not only determine how cells respond to metal-induced stress, but also define their vulnerability to different modes of cell death 134, 135.

Building on this perspective, the framework enables concrete predictions regarding execution trajectories and intervention sensitivities across disease contexts. In cancer, for example, many cells exhibit increased reliance on mitochondrial metabolism and elevated TCA cycle flux, which predisposes them to cross lipoylation-associated stress thresholds under increased copper burden. As a result, these cells are more likely to display heightened sensitivity to cuproptosis-like mechanisms. In contrast, tumor subtypes characterized by extensive lipid remodeling and enrichment of polyunsaturated fatty acids are more prone to enter ferroptotic execution pathways under conditions of iron overload or oxidative stress. These observations suggest that distinct metabolic subtypes of tumors may correspond to different metal-dependent vulnerability profiles, thereby providing a conceptual basis for stratified therapeutic interventions.

A similar predictive logic applies to neurodegenerative diseases. As disease progression advances, the gradual depletion of antioxidant defenses (e.g., GPX4-GSH systems), together with sustained mitochondrial dysfunction, renders neurons increasingly susceptible to lipid peroxidation driven damage, favoring ferroptosis-like outcomes. At the same time, in certain stages, mitochondrial proteostasis disruption and Fe-S cluster instability may coexist, indicating the presence of hybrid or transitional execution states. Such coexistence of multiple stress modalities may contribute to the complex and heterogeneous pathological features observed in neurodegeneration.

Importantly, these predictions are experimentally testable. Integrative analysis of transcriptomic, metabolomic, and lipidomic data can be used to stratify disease states or subtypes based on their position along the metallo-redox-metabolic axis. Subsequent perturbation strategies—such as modulating metal availability (e.g., copper or iron levels), targeting antioxidant systems (e.g., GPX4 activity), or reprogramming metabolic pathways (e.g., shifting between OXPHOS and glycolysis)—can then be employed to assess whether predicted vulnerability patterns are recapitulated. Furthermore, the integration of single-cell and spatial multi-omics approaches offers the opportunity to resolve spatiotemporal heterogeneity across cell populations within tissues, thereby providing a more comprehensive framework for validating disease-associated predictions.

#### Potential Execution Modes: Beyond Canonical Cuproptosis and Ferroptosis

The framework further suggests that additional, yet uncharacterized, metal-dependent execution modes may exist. Under alternative combinations of metal stress, metabolic wiring, and redox imbalance, cells may resolve stress through biochemical processes that differ from canonical cuproptosis or ferroptosis 136-139. These potential modes are expected to share common upstream regulatory architecture while exhibiting distinct downstream execution signatures. This perspective implies that currently defined forms of metal-dependent cell death may represent only a subset of observable endpoints within a broader execution landscape.

In summary, the unified metallo-redox-metabolic framework not only provides a coherent conceptual basis for interpreting cuproptosis and ferroptosis, but also endows metal-dependent cell death with predictive capacity at the systems level. By representing cellular states as dynamic configurations within a multidimensional variable space, the framework enables the formulation of biologically meaningful and experimentally testable hypotheses across multiple dimensions, including execution bias, threshold-dependent switching, disease-associated vulnerabilities, and potential execution modes. These observations collectively suggest that metal-dependent cell death arises not from isolated pathway activation, but from system-level responses when multilayered regulatory networks cross critical thresholds. The key predictions, representative measurable variables, and potential validation strategies are summarized in Table 4. Importantly, these insights further point toward a shift in research paradigm—from pathway-centric mechanistic dissection toward system-level modeling and multi-scale validation based on cellular state configurations. In this context, the integration of multi-omics data, computational modeling, and targeted perturbation strategies will be essential for dynamically characterizing system states and rigorously testing these predictions 140, 141, which will be further discussed in the following section.

## A Framework-Driven Strategy for State Representation, Computation, and Validation of Metal-Dependent Cell Death

Metal-dependent cell death is not governed by isolated molecular pathways but emerges from the coordinated interplay among metal homeostasis, mitochondrial metabolism, and redox regulation. Within this context, cell fate is more appropriately interpreted as a state-dependent process, defined by a cell's position and dynamic trajectory along the metal-metabolism-redox vulnerability axis. This positioning is reflected in continuous biases toward distinct execution modes, as well as threshold-dependent transitions between adaptive and irreversible states. Building on this conceptual foundation, the unified metal-metabolism-redox framework integrates cuproptosis and ferroptosis within a shared regulatory space, thereby providing a systematic perspective for understanding both their common principles and mechanistic divergences.

In parallel, recent advances in multi-omics technologies have enabled high-dimensional characterization of cellular states across diverse physiological and pathological conditions 142, 143. At the same time, the rapid development of artificial intelligence and machine learning has markedly enhanced the capacity to model complex nonlinear biological systems. Moreover, innovations in gene editing, metabolic perturbation, and high-resolution experimental manipulation have made it increasingly feasible to dissect causal regulatory mechanisms under controlled conditions. Together, these developments provide essential technical and conceptual support for investigating metal-dependent cell death at a systems level.

**Figure 6:**
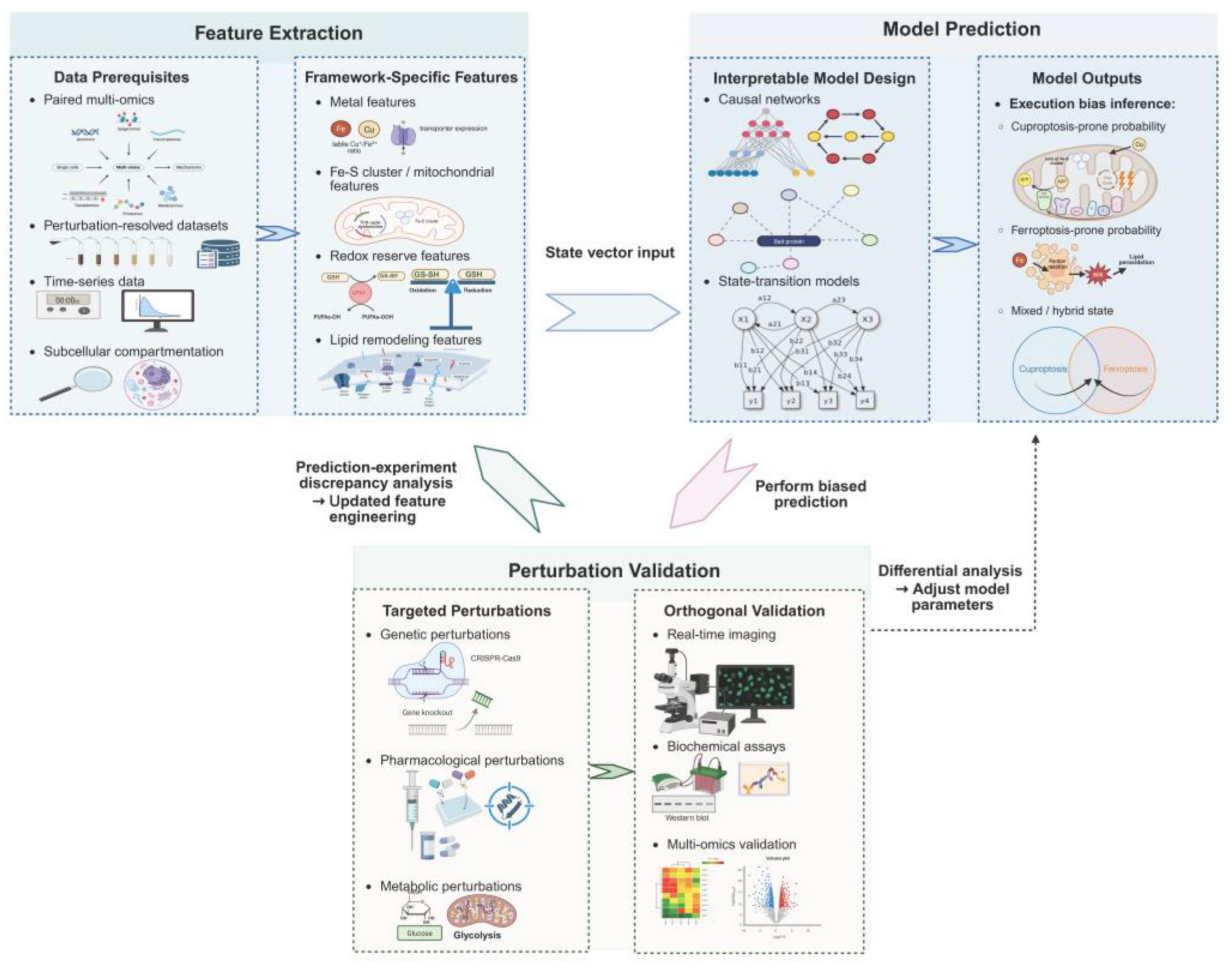
** A dry-wet iterative validation loop for the unified framework.** The loop integrates multi-omics profiling, computational modeling, and experimental perturbation with iterative feedback refinement. Three core components form a closed loop: (i) Data integration and state profiling: Multi-omics data (transcriptomics, proteomics, metabolomics, lipidomics) are integrated into state vectors representing metal handling, metabolic load, and redox buffering. (ii) Model training and prediction: Multimodal learning models map cellular states to execution bias predictions: cuproptosis-prone, ferroptosis-prone, or mixed probabilities. (iii) Experimental validation and mechanistic dissection: Targeted perturbations (metal loading, metabolic reprogramming, antioxidant intervention) with orthogonal readouts test model predictions. Feedback refinement: Prediction-experiment discrepancy analysis drives iterative updates to feature engineering, model parameters, and framework boundaries.

On this basis, a shift from traditional pathway-centric studies toward a framework-driven integrative research paradigm becomes both necessary and timely. In such a paradigm, multi-omics data are used to define cellular state distributions within the vulnerability space; computational modeling is employed to infer execution bias and state transition dynamics; and experimental perturbations are designed to interrogate key dimensions of the framework and validate their causal effects on system behavior. Importantly, experimental observations can be iteratively incorporated to refine both the computational models and the framework itself, thereby establishing a closed-loop research strategy centered on continuous validation and refinement (Fig. 6). Within this system, diverse data types and methodological approaches collectively contribute to evaluating and extending the explanatory power of the proposed framework.

### Multi-Omics Driven Representation of Cellular State Space

Within the unified metal-metabolism-redox framework, the value of multi-omics data lies not merely in providing layered molecular information, but in enabling the coordinated characterization of cellular states under metal stress conditions. Rather than serving as complementary resources for dissecting isolated mechanisms, multi-omics integration in this context aims to transform variations in metal handling, metabolic load, redox buffering, and latent execution propensity into observable, comparable, and model-compatible state descriptors. This representation allows metal-dependent cell death to be conceptualized not as a collection of discrete mechanisms, but as a dynamic process unfolding within a continuous state space 144, 145.

From the internal logic of the framework, different omics layers contribute distinct yet interrelated aspects of state representation. Transcriptomic and proteomic data primarily reflect the molecular preparedness of cells in terms of metal handling, mitochondrial metabolism, and redox regulation. For instance, the expression of metal transporters, buffering systems, lipoylation-related enzymes, Fe-S cluster assembly factors, lipid remodeling enzymes, and antioxidant regulators collectively defines the molecular basis of the metal-handling, metabolic integration, and redox-buffering components, thereby shaping the latent susceptibility of cells prior to and during stress accumulation 146, 147.

By contrast, metabolomic and lipidomic data capture functional features that more directly influence execution bias. Metabolomics provides insights into tricarboxylic acid cycle activity, oxidative phosphorylation dependence, and metabolic reprogramming, thereby reflecting the metabolic burden and mitochondrial stress under metal exposure 148. Lipidomics characterizes the abundance of polyunsaturated phospholipids and the availability of substrates for lipid peroxidation, which are critical determinants of membrane vulnerability. Together, these features form a key bridge between the metabolic and execution layers, offering direct indicators of the preferred execution trajectory.

Redox-related features constitute another indispensable component of state representation. ROS levels, GSH reserves, GPX4-associated antioxidant capacity, and the accumulation of lipid peroxidation products collectively define the redox buffering status of the cell and its proximity to destabilization thresholds. Within the framework, the redox layer functions as a dynamically coupled regulatory module, continuously interacting with metal handling and metabolic processes. Consequently, redox measurements are not limited to assessing oxidative stress, but also serve to identify transitions from adaptive states toward execution commitment 149-151.

At a higher level, the central task of multi-omics integration is to organize these heterogeneous features into unified state vectors and to construct a state space that captures cellular heterogeneity and dynamic transitions 152. In this space, cellular states are represented as continuous distributions rather than discrete categories, providing a quantitative basis for understanding gradual shifts in cell fate. Such continuous representations enable the identification of latent execution tendencies across different cellular contexts.

Building on this representation, multi-omics feature combinations can be abstracted into discriminative state patterns that define execution propensity within the framework. For example, a state characterized by enhanced copper accumulation, increased mitochondrial lipoylated protein burden, elevated tricarboxylic acid cycle activity, and relatively preserved redox buffering capacity can be summarized as a “cuproptosis-prone” signature. In contrast, a state marked by iron overload, enrichment of polyunsaturated phospholipids, glutathione depletion, and accumulation of lipid peroxidation products constitutes a representative “ferroptosis-prone” signature. Importantly, these signatures reflect coordinated cross-omics patterns rather than isolated features, capturing the systemic balance among metal handling, metabolic configuration, and redox buffering.

Furthermore, temporal and spatial resolution are critical for accurately characterizing state dynamics. Metal-dependent cell death is inherently a time-evolving process, and single time-point measurements cannot capture continuous state transitions. Time-resolved multi-omics analyses facilitate the reconstruction of state trajectories 153, while single-cell and spatial omics approaches reveal heterogeneity across cell populations and microenvironmental niches 154, 155. Such spatiotemporal insights are particularly important for understanding disease contexts characterized by pronounced cellular diversity.

Despite these advantages, multi-omics-based state characterization faces practical challenges, including data heterogeneity, differences in measurement scales, and difficulties in cross-platform integration. Therefore, within a framework-driven paradigm, effective integration requires the establishment of interpretable and comparable feature systems centered on key dimensions such as metal handling, metabolic integration, and redox buffering. Only under such structured organization can multi-omics data transition from descriptive resources to actionable inputs for computational modeling and experimental validation.

Overall, multi-omics integration organizes heterogeneous molecular features into a unified state representation that captures cellular variability and dynamic transitions under metal stress. This structured representation enables the systematic extraction of execution-related features and provides a coherent basis for subsequent computational modeling of cellular states and fate decisions.

### Computational Modeling of State Space and Execution Bias

Building upon the multi-omics defined cellular state space, computational modeling serves to quantitatively evaluate the global configuration of cells along the metal-metabolism-redox vulnerability axis, transforming high-dimensional molecular information into interpretable system-level behavior 156-158. Through continuous representation and modeling of this state space, it becomes possible to infer the relative positioning of cells across different execution modes and to predict their potential trajectories, thereby converting cell fate determination from a static description into a computable dynamic process 159.

Within this framework, modeling is not confined to a single predictive task but instead derives multiple biologically meaningful analytical dimensions from the underlying state representation. Execution bias describes the relative positioning of cells across alternative metal-dependent death modalities; threshold-related features capture the critical conditions under which cells transition from adaptive states to irreversible execution; and global state assessment integrates metal handling capacity, metabolic load, and redox buffering to evaluate overall cellular stability and vulnerability. Together, these dimensions provide a multidimensional quantitative characterization of cell fate decisions and establish a basis for mechanistic interpretation.

At the level of computational implementation, multi-omics data are first organized into a unified state representation, enabling heterogeneous molecular information to be integrated within a common feature space. Transcriptomic, proteomic, metabolomic, and lipidomic data are standardized and aligned to generate high-dimensional vectors that capture cellular characteristics across key biological dimensions 160. This unified representation preserves cross-omics relationships while providing structured inputs for downstream modeling. To adequately cover the state space, such datasets typically encompass diverse metal exposure conditions, metabolic perturbations, and redox challenges, and may incorporate temporal resolution and subcellular localization where available.

Feature construction is centered on core dimensions of the framework. Metal-related features quantify the handling and buffering of copper and iron; metabolic features reflect mitochondrial activity, Fe-S cluster stability, and pathway dependencies; redox features capture antioxidant capacity and oxidative stress levels; and lipid-related features assess membrane susceptibility to peroxidation. Together, these features form a multidimensional embedding of cellular states, allowing models to capture coordinated biological processes rather than isolated variables.

In terms of model construction, multimodal modeling strategies built upon cell foundation models provide an effective route for state computation. Pre-trained on large-scale single-cell transcriptomic data, such models are capable of learning generalizable representations of cellular states. On this basis, they can be further extended by integrating proteomic, metabolomic, and lipidomic information, enabling a unified multimodal representation of cellular states. Moreover, the incorporation of structured modeling approaches, such as graph neural networks (GNNs), allows the explicit characterization of dependencies and interactions among different state variables, particularly across key processes including metal handling, mitochondrial metabolism, and redox regulation. In parallel, multimodal deep learning frameworks facilitate the integration of heterogeneous molecular layers within a shared representation space, thereby enhancing the capacity to capture the global structure of complex state landscapes.

From a dynamic perspective, the introduction of temporal modeling strategies or pseudotime inference based on single-cell data enables the reconstruction of cellular trajectories within the state space, allowing the identification of critical transition points and potential thresholds that govern state shifts. Coupled with task-specific fine-tuning, these models can simultaneously support multiple predictive objectives within a unified representation framework, including the estimation of execution bias, analysis of state transitions, and identification of key regulatory variables. Importantly, while retaining strong generalization capability, such modeling strategies are able to capture both coordinated variations across molecular layers and their dynamic evolution, thereby providing a robust computational foundation for the systematic characterization of complex cellular state spaces.

Model outputs are typically expressed as continuous variables that quantitatively describe cellular states and their evolution. Execution bias can be represented as probabilities or scoring functions reflecting relative tendencies toward different execution modes. State transitions can be characterized through trajectory inference or transition probabilities, revealing dynamic movement within the state space. Critical thresholds can be identified through sensitivity analyses of state perturbations, pinpointing key factors that drive system-level transitions. Collectively, these outputs transform otherwise inaccessible system behaviors into analyzable and comparable computational entities.

Through this modeling framework, artificial intelligence enables the transformation of multi-omics defined state spaces into computable analytical systems, allowing both the positioning of cells along the vulnerability axis and their dynamic trajectories to be quantitatively assessed. This not only provides new insights into the regulatory mechanisms of metal-dependent cell death but also offers precise guidance for experimental interrogation of critical states and transition points, thereby tightly linking computational analysis with mechanistic validation 161-163.

### State Perturbation-Driven Experimental Validation and Mechanistic Dissection

Following computational modeling, experimental systems are required to directly validate predicted cellular states along the vulnerability axis and to identify the key determinants driving state transitions. Unlike traditional approaches centered on validating individual molecular functions, experimental design within this framework focuses on system-level state dynamics, emphasizing how controlled perturbations reshape cellular behavior and translate computational predictions into observable biological responses 164-166.

Experimentally, the design typically follows a “state perturbation-system response” paradigm. Perturbations target key dimensions of the vulnerability axis, including metal ion availability, mitochondrial metabolic activity, and redox balance. For example, modulation of copper or iron levels directly alters the metal-handling component; manipulation of tricarboxylic acid cycle activity or mitochondrial function reshapes metabolic load; and interventions targeting glutathione metabolism or reactive oxygen species influence redox buffering capacity. These perturbations are not applied in isolation but are used to drive cells across different regions of the state space, thereby enabling direct testing of computational predictions 167, 168.

Correspondingly, experimental readouts must capture system responses across multiple dimensions to accurately reflect execution modes. Multi-parametric measurements—such as lipid peroxidation levels, protein aggregation, mitochondrial alterations, cell viability, and functional outputs—are typically combined to provide an integrated assessment of cellular outcomes. This multidimensional strategy reduces the bias associated with single markers and ensures that experimental observations align with the state-based representations used in computational modeling.

Different types of predictive outputs can be validated through tailored experimental designs. Execution bias can be assessed by applying standardized perturbations to cells in comparable initial states and evaluating the relative activation of distinct execution markers. Threshold identification can be achieved by gradually increasing perturbation intensity or duration and identifying transition points from reversible adaptation to irreversible execution. Global state assessment can be examined by comparing responses to identical perturbations across different physiological or pathological contexts, thereby revealing how baseline configurations influence fate decisions. In more complex settings, combinatorial readouts can also be used to identify potential interactions between distinct execution pathways.

To further resolve state transitions, temporal dynamics must be incorporated into experimental design. Time-resolved measurements enable the distinction between early adaptive responses and later irreversible execution phases and reveal the sequential contributions of regulatory factors during state evolution. Such dynamic analyses are essential for understanding how execution bias emerges and how thresholds are crossed over time 169-171.

Overall, this state-centric experimental strategy allows computational predictions to be validated at the systems level while providing direct entry points for mechanistic investigation. By aligning perturbation strategies with state dimensions and integrating multi-layered readouts, complex cellular behaviors can be interpreted in terms of coordinated system states rather than isolated pathways.

In addition, experimentally derived response data can be incorporated to refine computational models. By comparing observed outcomes with predicted states, discrepancies can be identified and used to adjust model parameters or feature representations, improving predictive accuracy. This iterative integration of computation and experimentation establishes a stable feedback loop, ultimately enabling a progressively refined understanding of metal-dependent cell death mechanisms 172.

## Conclusions and perspectives

Traditionally, copper- and iron-dependent forms of cell death have been investigated as distinct molecular processes, each driven by specific metal ions and their associated biochemical pathways. However, the integrated mechanistic, multi-omics, and disease-related evidence synthesized in this review supports a fundamentally different view: these two forms of cell death arise from a shared regulatory foundation, defined by a continuous state space shaped by metal homeostasis, cellular metabolic configuration, and redox balance. Within this framework, cuproptosis and ferroptosis are no longer interpreted as isolated pathways, but rather as distinct execution outcomes corresponding to different regions of this state space 170, 173. This perspective shifts the understanding of metal-dependent cell death from a collection of parallel mechanisms to a state-driven process of cellular fate determination.

Under this unified view, cellular fate is governed by a set of quantifiable state variables with clear biological interpretations. These include the integrity of Fe-S clusters, mitochondrial metabolic flux and its responsiveness to energetic demands, the composition and peroxidation susceptibility of polyunsaturated fatty acids in membrane lipids, and the overall redox buffering capacity of the cell. The coupling among these variables defines the execution bias of cells under metal-induced stress 174, determining whether cells preferentially undergo cuproptosis, characterized by proteotoxic aggregation, or ferroptosis, driven by lipid peroxidation 175, 176. Importantly, the continuous variation and interaction of these state variables further define critical thresholds and trajectories governing transitions between different modes of cell death, enabling cellular fate to be described as a continuous process within a unified state space rather than as discrete events 177, 178.

Building upon this conceptual foundation, the study of metal-dependent cell death can be advanced from mechanistic characterization toward a framework centered on state computation and prediction. By integrating multi-layered data, including transcriptomics, proteomics, metabolomics, and lipidomics, cells can be mapped into high-dimensional state vectors, allowing their positions and dynamic trajectories within the state space to be computationally modeled. Artificial intelligence provides essential methodological support for this process. In particular, pre-trained cell representation models can learn generalizable embeddings of cellular states, while structured models such as graph neural networks enable the characterization of dependencies among key state variables. Multimodal learning frameworks further facilitate the integration of heterogeneous molecular layers into a unified representation. In addition, temporal modeling strategies and pseudotime inference approaches can be employed to reconstruct cellular trajectories, enabling the identification of critical transition points and potential thresholds that govern state shifts. Within this framework, model outputs extend beyond binary classification of cell death modalities, instead providing continuous measures such as execution bias scores, transition probabilities, and sensitivity to perturbations, thereby rendering cellular fate decisions computationally tractable and predictive 179-182.

Importantly, this computational framework is inherently testable and can be systematically evaluated through experimental strategies. For instance, the functional status of Fe-S clusters can be assessed through enzyme activity measurements or stability analyses of iron-sulfur proteins, allowing validation of their role in coupling metal load to metabolic stress. Mitochondrial metabolic flux and ROS production can be dynamically quantified using metabolic flux analysis and redox-sensitive probes, providing insight into their roles in driving state transitions. Similarly, lipid peroxidation levels and antioxidant system capacity can be measured to evaluate cellular susceptibility to ferroptosis. These experimental readouts not only enable validation of model predictions regarding key state variables, but also allow targeted perturbations—such as modulation of metal transport, mitochondrial function, or antioxidant systems—to test predicted transition pathways, thereby supporting a falsifiable and experimentally grounded modeling framework.

Furthermore, this framework naturally supports a closed-loop research paradigm integrating computation, experimentation, and iterative refinement 183. Computational models generate predictions regarding key regulatory nodes and transition thresholds based on multi-omics data, experimental systems validate these predictions through systematic perturbations, and the resulting data are fed back into model optimization and structural refinement 183. This iterative strategy enhances both predictive performance and biological interpretability, facilitating a shift from static description toward dynamic modeling and predictive control of metal-dependent cell death processes.

From a translational perspective, conceptualizing cell death as a function of position and transition within a state space provides new principles for therapeutic intervention. Rather than directly targeting terminal execution mechanisms, modulating upstream state variables to reshape the distribution of cellular states may offer a more robust and selective strategy. For example, in cancer contexts, increasing copper load and mitochondrial metabolic stress may shift cells toward a cuproptosis-prone region, while concomitant suppression of antioxidant defenses may enhance ferroptosis susceptibility, enabling synergistic cell killing. In contrast, in neurodegenerative diseases, stabilizing metal homeostasis, enhancing redox buffering capacity, and maintaining metabolic balance may prevent cells from transitioning toward irreversible damage states. Such state-oriented intervention strategies offer a conceptual framework for precision targeting of metal-associated pathologies 184, 185.

Despite these advances, several key challenges remain. First, the integration of multi-omics data across temporal and spatial dimensions remains limited, particularly in capturing continuous state transitions at single-cell and subcellular resolution. Second, the generalizability of current models across tissues and disease contexts requires further validation. Third, maintaining biological interpretability while increasing model complexity remains a critical challenge for future development.

In summary, the unified framework proposed in this review not only integrates the mechanistic understanding of cuproptosis and ferroptosis, but also elevates the field toward a system-level paradigm characterized by computability, predictability, and experimental testability. With continued advances in multi-omics data generation, artificial intelligence modeling, and system-level experimental validation, it will become increasingly feasible to construct predictive models of cellular state and fate. Such models hold promise for applications in disease stratification, risk assessment, and precision therapy, ultimately enabling a transition from mechanistic insight to predictive and actionable biomedical strategies.

## Figures and Tables

**Table 1:** Comparative mechanistic features of cuproptosis and ferroptosis within the metal-metabolism-redox vulnerability axis

Category	Cuproptosis	Ferroptosis	Shared/Integrative insight
Triggering metal species	Reduced copper (Cu⁺)	Ferrous iron (Fe²⁺)	Metal dyshomeostasis provides the initiating stress input
Primary entry/accumulation context	Copper ionophores; mitochondrial copper accumulation; copper reduction by FDX1	Transferrin-TFR1-mediated iron uptake; expansion of the labile iron pool	Dysregulated metal import, buffering, and intracellular partitioning shape susceptibility
Primary biochemical insult	Copper-induced aggregation of lipoylated mitochondrial proteins and proteotoxic stress	Iron-catalyzed phospholipid peroxidation	Distinct toxic chemistries arise from redox-active metals
Core execution substrate	Lipoylated mitochondrial protein complexes	PUFA-containing membrane phospholipids	Execution specificity is determined by the dominant vulnerable substrate
Dominant execution mode	Mitochondrial proteotoxic collapse	Membrane failure driven by lipid peroxidation	Distinct execution branches emerging from a shared stress landscape
Metabolic dependency: bioenergetic module	Strong dependence on mitochondrial TCA-cycle activity and oxidative phosphorylation	Bioenergetic metabolism modulates ROS-generating pressure and ferroptotic threshold	Mitochondrial metabolic load amplifies metal-induced stress
Metabolic dependency: lipid remodeling module	Not a primary execution determinant	ACSL4/LPCAT3-dependent incorporation of PUFA into membrane phospholipids determines peroxidation susceptibility	Lipid remodeling is specifically critical for ferroptotic propagation
Fe-S cluster involvement	Fe-S related dysfunction may affect lipoylation-linked enzymes, respiratory proteins, and cluster-maintenance factors, thereby amplifying mitochondrial collapse	Oxidative damage to metabolically and respiratorily relevant Fe-S proteins can impair TCA flux and increase labile iron availability	Fe-S proteins function as heterogeneous coupling nodes rather than a single uniform convergence point
Redox features	Secondary ROS accumulation accompanying proteotoxic and bioenergetic failure	ROS participate directly in initiation and propagation of lipid peroxidation	Redox imbalance sensitizes both pathways, but with different mechanistic roles
Antioxidant control	GPX4-independent execution; thiol-dependent redox buffering (including the GPX4-GSH system) may indirectly modulate susceptibility	Strong dependence on the GSH-GPX4 axis for detoxification of lipid hydroperoxides	Antioxidant buffering influences both pathways, but GPX4 is a core executor only in ferroptosis
Subcellular site of failure	Mitochondrial matrix and inner mitochondrial metabolic machinery	Cellular and organelle membranes	Spatial context contributes to phenotypic divergence
Morphological outcome	Mitochondrial condensation, matrix proteotoxicity, and metabolic collapse	Membrane rupture, lipid damage, and necrotic-like morphology	Both are non-apoptotic cell death phenotypes
Representative pathway regulators	FDX1, protein lipoylation machinery, mitochondrial copper handling systems	System Xc⁻, GPX4, ACSL4, LPCAT3, iron handling pathways	Partial overlap occurs through upstream redox and metabolic regulation
Typical pharmacological modulation	Copper chelators, copper ionophores, modulation of mitochondrial copper stress	Ferroptosis inhibitors (e.g., ferrostatins, liproxstatin-1), GPX4/system Xc⁻ targeting	Therapeutic responses may converge when upstream vulnerability states are co-perturbed

**Table 2:** Cross-disease configurations of the metal-metabolism-redox vulnerability axis and associated execution modes

Axis dimension	Configuration spectrum	Representative contexts	Functional implication
Metal homeostasis	Adaptive utilization ↔ Dysregulated accumulation	Proliferative tumors; stressed neurons; degenerative tissues	Metal imbalance as a primary upstream stressor
Mitochondrial state	Metabolic flexibility ↔ Energetic failure	Hypermetabolic cells; therapy-stressed states; neurodegeneration	Governs stress amplification and energy resilience
Fe-S cluster integrity	Functional coupling ↔ Structural destabilization	Rapid proliferation; oxidative stress; aging tissues	Integrates metal, metabolic, and redox signals
Redox buffering	Compensated balance ↔ Antioxidant exhaustion	Adaptive stress responses; chronic pathology	Defines threshold between adaptation and damage
Axis positioning	Dynamic continuum (stable ↔ vulnerable states)	Stage-, cell type-, and microenvironment-dependent	Determines overall cellular vulnerability landscape
Execution mode bias	Context-dependent engagement	Variable across diseases and stages	Cuproptosis-prone; ferroptosis-prone; mixed states

**Table 3:** Functional components, inner communication, and system-level roles within the unified metallo-redox-metabolic framework

Framework component	Core role in the system	Representative nodes/processes	Major incoming/outgoing signals	Contribution to system instability and execution bias
Metal-handling component	Establishes intracellular metal availability and pressure landscape, providing the primary stress input to the system	Metal transporters and exporters (e.g., SLC31A1, ATP7A/B, TFR1), ferritin, metallochaperones, labile Cu⁺ and Fe²⁺ pools, metal sequestration and chelation systems	Outgoing: altered metal cofactor binding, mitochondrial metal loading, Fe-S cluster perturbation; Feedback from downstream: redox-dependent modulation of transporter activity and metal redistribution	Shapes basal susceptibility and initiates upstream stress, determining initial positioning along the vulnerability axis
Metabolic integration component	Integrates and amplifies metal-derived stress through mitochondrial bioenergetics and lipid metabolic remodeling	TCA cycle, oxidative phosphorylation (OXPHOS), Fe-S cluster biogenesis and maintenance, lipoylation-related processes, ACSL4, LPCAT3, PUFA-phospholipid remodeling	Incoming: metal imbalance and altered cofactor availability; Outgoing: ROS accumulation, NADPH depletion, increased thiol demand, substrate-specific vulnerability (mitochondrial vs membrane)	Determines how stress is amplified and distributed, biasing instability toward mitochondrial proteotoxicity or lipid peroxidation
Redox buffering component	Maintains and dynamically adjusts intracellular redox balance, acting as a central regulator of system stability and reversibility	GSH-GPX4 axis, thiol-dependent redox networks, NADPH-dependent antioxidant systems, lipid peroxide detoxification pathways	Incoming: ROS accumulation, metabolic strain; Outgoing: regulation of metal transporter states, modulation of metabolic enzyme activity, control or failure of lipid peroxide detoxification	Sets the threshold between adaptive response and irreversible damage, governing the transition toward execution
Execution component	Converts multilayered stress into terminal cell death phenotypes through structural and biochemical collapse	Lipoylated mitochondrial proteins, Fe-S cluster-dependent enzymes, PUFA-containing membrane phospholipids, proteotoxic aggregates, lipid peroxidation modules	Incoming: mitochondrial proteotoxic stress, lipid peroxide accumulation, thiol collapse; Outgoing: ROS amplification, organelle dysfunction, secondary metal redistribution	Produces cuproptosis-biased, ferroptosis-biased, or mixed execution outcomes depending on dominant vulnerability substrates

**Table 4:** Framework-derived predictions and their experimentally testable features

Prediction	Representative measurable variables	Supporting data modalities	Illustrative perturbation strategies	Expected system-level patterns
Execution bias	DLAT lipoylation level, PUFA-PL abundance, GPX4 activity, labile Cu/Fe pools	Steady-state multi-omics, enzyme activity assays	Modulation of metal availability combined with metabolic state reprogramming (e.g., OXPHOS vs. glycolysis, PUFA availability)	System state-dependent bias, with mitochondrial proteotoxic stress favoring cuproptosis-like outcomes and lipid peroxidation favoring ferroptosis-like outcomes
Threshold-dependent switching	Thiol redox status, Fe-S cluster stability, cumulative ROS burden	Time-resolved redox proteomics and lipidomics	Sustained or evolving metal stress with temporally controlled intervention	Temporal transition of dominant execution mode as system bottlenecks shift from proteostasis to lipid redox control
Disease-specific vulnerability states	Metabolic dependency (OXPHOS vs glycolysis), PUFA composition, GPX4/GSH status, mitochondrial integrity, labile metal pools	Integrative multi-omics, clinical cohort data, single-cell and spatial omics	Modulation of metal levels, antioxidant systems, and metabolic pathways in disease-relevant models	Disease- or subtype-specific sensitivities to cuproptosis- or ferroptosis-like mechanisms, enabling stratified intervention strategies
Latent or hybrid execution modes	Coexistence of protein aggregation and lipid peroxidation signatures	Spatially resolved multi-omics	Combined perturbation of metal stress and antioxidant systems	Emergence of mixed or intermediate phenotypes reflecting coupled proteotoxic and membrane damage processes

## Abbreviations

PCD: Programmed cell death
Fe-S: iron-sulfur
TCA: Tricarboxylic acid
ROS: Reactive oxygen species
ATP: Adenosine triphosphate
PUFA-PL: Polyunsaturated Fatty Acid-Phospholipid
GPX4: Glutathione Peroxidase 4
GSH: Glutathione
PUFAs: Polyunsaturated phospholipids
OXPHOS: oxidative phosphorylation
ETC: electron transport chain
·OH: hydroxyl radicals
PL-PUFAs: phospholipid-bound polyunsaturated fatty acids
PUFAs-OOH: PUFA hydroperoxides
